# The complete mitochondrial genome of the house dust mite *Dermatophagoides pteronyssinus *(Trouessart): a novel gene arrangement among arthropods

**DOI:** 10.1186/1471-2164-10-107

**Published:** 2009-03-13

**Authors:** Wannes Dermauw, Thomas Van Leeuwen, Bartel Vanholme, Luc Tirry

**Affiliations:** 1 Department of Crop Protection, Faculty of Bioscience Engineering, Ghent University, Coupure links 653, B-9000, Ghent, Belgium; 2 Department of Molecular Biotechnology, Faculty of Bioscience Engineering, Ghent University, Coupure links 653, B-9000, Ghent, Belgium; 3 VIB Department of Plant Systems Biology, Ghent University, Technologiepark 927, B-9052, Ghent, Belgium

## Abstract

**Background:**

The apparent scarcity of available sequence data has greatly impeded evolutionary studies in Acari (mites and ticks). This subclass encompasses over 48,000 species and forms the largest group within the Arachnida. Although mitochondrial genomes are widely utilised for phylogenetic and population genetic studies, only 20 mitochondrial genomes of Acari have been determined, of which only one belongs to the diverse order of the Sarcoptiformes. In this study, we describe the mitochondrial genome of the European house dust mite *Dermatophagoides pteronyssinus*, the most important member of this largely neglected group.

**Results:**

The mitochondrial genome of *D. pteronyssinus *is a circular DNA molecule of 14,203 bp. It contains the complete set of 37 genes (13 protein coding genes, 2 rRNA genes and 22 tRNA genes), usually present in metazoan mitochondrial genomes. The mitochondrial gene order differs considerably from that of other Acari mitochondrial genomes. Compared to the mitochondrial genome of *Limulus polyphemus*, considered as the ancestral arthropod pattern, only 11 of the 38 gene boundaries are conserved. The majority strand has a 72.6% AT-content but a GC-skew of 0.194. This skew is the reverse of that normally observed for typical animal mitochondrial genomes. A microsatellite was detected in a large non-coding region (286 bp), which probably functions as the control region. Almost all tRNA genes lack a T-arm, provoking the formation of canonical cloverleaf tRNA-structures, and both rRNA genes are considerably reduced in size. Finally, the genomic sequence was used to perform a phylogenetic study. Both maximum likelihood and Bayesian inference analysis clustered *D. pteronyssinus *with *Steganacarus magnus*, forming a sistergroup of the Trombidiformes.

**Conclusion:**

Although the mitochondrial genome of *D. pteronyssinus *shares different features with previously characterised Acari mitochondrial genomes, it is unique in many ways. Gene order is extremely rearranged and represents a new pattern within the Acari. Both tRNAs and rRNAs are truncated, corroborating the theory of the functional co-evolution of these molecules. Furthermore, the strong and reversed GC- and AT-skews suggest the inversion of the control region as an evolutionary event. Finally, phylogenetic analysis using concatenated mt gene sequences succeeded in recovering Acari relationships concordant with traditional views of phylogeny of Acari.

## Background

Approximately 48,000 mite and tick species (Arthropoda: Chelicerata: Arachnida: Acari) [1,2] have been described to this day. Since the number of undescribed species is thought to be twenty-fold higher, this subclass is by far the most species-rich group among the Arachnida [2]. Acari diversified 400 million years ago and currently three major lineages are recognised: Opilioacariformes, Acariformes and Parasitiformes [2,3]. The Acariformes comprise two major groups, the Trombidiformes and Sarcoptiformes [2,4]. Two of the most prominent members of the Sarcoptiformes are the European house dust mite *Dermatophagoides pteronyssinus *(Trouessart, 1897) and the American house dust mite *Dermatophagoides farinae *(Hughes, 1961), both belonging to the family of the Pyroglyphidae (cohort Astigmata). Pyroglyphid mites are typical inhabitants of animal nests. In the human environment, they are mainly found in upholstery, textile floor covers and beddings, where they primarily feed on the skin scale fraction in house dust [5]. About 40 years ago, house dust mites were first recognized as one of the major sources of allergens in house dust [6]. The allergenic proteins are found in high concentrations in mite faeces, which, after drying and pulverizing, become airborne and can be inhaled. The presence of these allergens in sensitive persons is able to cause diseases like asthma, dermatitis and rhinitis [7,8]. In countries with a temperate climate, 6 to 35 per cent of the population is sensitive to house dust mite-derived allergens [9].

Complete mitochondrial (mt) genome sequences are becoming increasingly important for effective evolutionary and population studies. Mt genome sequences are not only more informative than shorter sequences of individual genes, they also provide sets of genome-level characters, such as the relative position of different genes, RNA secondary structures and modes of control of replication and transcription [10-13]. However, the applicability of mt genomes as a marker of highly divergent lineages is still controversial [14,15] and remains to be elucidated [16]. In addition, unravelling mt genomes can be of economic importance as well, since several chemical classes of pesticides target mt proteins. Well-known acaricides like acequinocyl and fluacrypyrim affect mt electron transport through the inhibition of the mt encoded cytochrome b in complex III [17]. Also, the economically important class of METI (Mitochondrial Electron Transfer Inhibitors)-acaricides target the mt complex I, although their exact molecular target has not yet been elucidated. Recently, resistance to the acaricide bifenazate was shown to be caused by mutations in the mt encoded cytochrome b and to have evolved rapidly through a short stage of mt heteroplasmy [18].

At present, the mt genomes of 20 species belonging to the Acari are available at NCBI ([19], status January 10, 2009). Most of the submitted sequences have the typical features of metazoan mt genomes. They are circular, between 13 and 20 kb in length, contain a coding region with 37 genes (22 tRNAs, 2 rRNAs and 13 protein coding genes) and a relatively small non-coding region. The latter is mostly AT-rich and fulfils a role in the initiation of replication and transcription [20,21]. Compared to this typical configuration, the mt genomes of *Steganacarus magnus*, *Metaseiulus occidentalis *and *Leptotrombidium pallidum *show some abnormal features. *S. magnus *lacks 16 of the 22 tRNAs normally present in mt genomes [22]. *M. occidentalis *has a unusually large mt genome (24.9 kb) resulting from a duplication event of a large fragment of the codon region. Despite its large size, genes coding for nad6 and nad3 were not found during the initial annotation process [23]. *L. pallidum *on the other hand has 38 mt genes due to a duplication of the *16S-rRNA *[24].

In this study, we analyse the complete mt genome of a member of the Sarcoptiformes, the European house dust mite *D. pteronyssinus*, after obtaining the complete sequence using a long PCR approach.

## Results and discussion

### Genome organisation

**Figure 1:**
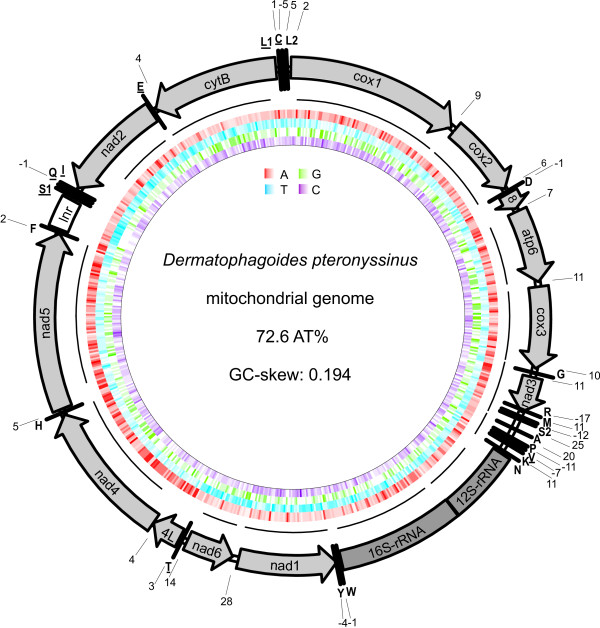
**Schematic representation of the mt genome of *D. pteronyssinus***. Except for *atp8 *(= 8) and *nad4 *(= 4L) protein coding and ribosomal genes are presented as outlined in the abbreviations section. tRNA genes are abbreviated using the one-letter amino acid code, with L_1 _= CUN; L_2 _= UUR; S_1 _= AGN; S_2 _= UCN. RNAs on the N-strand are underlined. Numbers at gene junctions indicate the length of small non-coding regions where negative numbers indicate overlap between genes. A-,T-,G- and C-content of the mt genome is represented using a red, blue, green and purple colour graded circle, respectively. Black curved lines on the outside of these circles represent mt genome coverage by *Dermatophagoides *ESTs (see additional file 5 for sequences of *Dermatophagoides *ESTs covering the mt genome of *D. pteronyssinus*).

**Figure 2:**
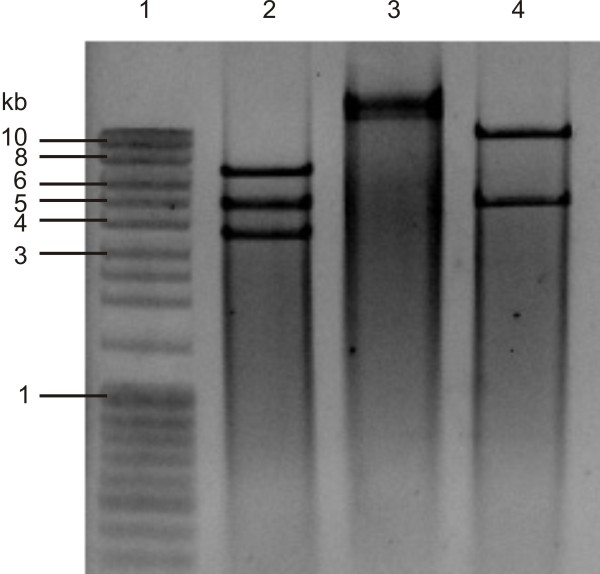
**Restriction digest of rolling circle amplified mitochondrial DNA of *D. pteronyssinus***. Rolling circle amplified mtDNA, undigested (lane 3) and digested with *Xmn*I (lane2) and *Eco*RI (lane 4). Molecular marker used was MassRuler DNA ladder Mix (Fermentas) (lane 1).

The mt genome of *D. pteronyssinus *was amplified, using long PCR, in three overlapping fragments. The final assembled sequence was 14,203 bp [GenBank: EU884425; Fig. 1], making it the fifth smallest sequenced genome within the Acari. Only the mt genomes of *Tetranychus urticae *(13,103 bp), *Leptotrombidium akamushi *(13,698 bp), *Leptotrombidium deliense *(13,731 bp) and *S. magnus *(13,818 bp) are smaller (Table 1 [25-27]). As non-specific amplification artefacts and incomplete coverage of genes are well-known drawbacks of a PCR approach [28], we checked the genome size by restriction digest on rolling circle amplified mtDNA (Fig. 2). This approach confirmed the sequence size, considering that the relative mobility of mtDNA restriction fragments can show slight (5–12%) deviations compared to their sequence length [29]. The mt genome of *D. pteronyssinus *is the first mt sequence of a mite belonging to the Astigmata and is together with the mt genome of *S. magnus *the only representative from the order of the Sarcoptiformes. Adding this genome to the database resulted in 21 publicly available Acari mtDNA sequences. Twelve belong to species in the superorder of the Parasitiformes whereas nine – among which *D. pteronyssinus *– belong to species in the superorder of the Acariformes.

**Table 1:** Nucleotides composition of completely sequenced mt genomes of Acari and Limulus polyphemus*.

**Organism **^a^	**S**^b^	**Genbank acc. nr**	**Complete mt genome**				**mt PCG**^c^		**12S-rRNA**		**16S-rRNA**		**Control Region(s)**^d^		**Ref**. ^e^
			Length (bp)	AT%	AT-skew^f^	GC-skew^f^	Length (bp)	AT%	Lengh (bp)	AT%	Length (bp)	AT%	Length (bp)	AT%	
*D. pteronyssinus*	A	EU884425	14,203	72.60	-0.199	0.194	10,826	71.61	665	72.93	1078	76.07	286	91.61	this study
*Am. triguttatum*	P	NC_005963	14,740	78.40	-0.022	-0.133	10,876	78.29	693	79.65	1199	81.82	307-307	71.66-71.01	unpub.
*As. *sp.^g^	A	NC_010596	16,067	70.07	0.015	-0.049	10,560	69.27	680	72.79	1047	76.31	1207-1236	70.01–70.06	unpub.
*C. capensis*	P	NC_005291	14,418	73.54	0.036	-0.374	10,873	72.66	695	76.26	1225	78.29	342	71.35	[38]
*H. flava*	P	NC_005292	14,686	76.91	-0.018	-0.116	10,817	76.65	699	78.40	1196	81.61	310-310	66.45–66.77	[38]
*I. hexagonus*	P	NC_002010	14,539	72.66	0.033	-0.366	10,826	71.13	705	78.44	1287	72.60	359	71.87	[37]
*I. holocyclus*	P	NC_005293	15,007	77.38	-0.013	-0.254	10,860	76.38	716	77.93	1214	81.55	352–450	78.41–80.00	[26]
*I. persulcatus*	P	NC_004370	14,539	77.34	-0.024	-0.269	10,879	76.59	720	78.89	1206	79.77	352	77.56	[26]
*I. uriae*	P	NC_006078	15,053	74.79	0.007	-0.328	10,837	73.75	712	78.09	1210	78.35	388–476	77.06-74.16	[26]
*Le. akamushi*	A	NC_007601	13,698	67.47	-0.016	-0.075	10,292	67.19	596	67.11	1026	72.03	260–262	60.38-59.54	[74]
*Le. deliense*	A	NC_007600	13,731	69.95	-0.017	-0.058	10,292	70.06	602	70.27	1023	73.02	294–301	62.24-61.79	[74]
*Le. pallidum*^h^	A	NC_007177	16,779	70.96	-0.031	-0.044	10,312	71.38	601	72.05	1008	74.90	537-724-736-803	63.87-66.71-66.75-66.50	[24]
*M. occidentalis*^i^	P	NC_009093	24,961	75.97	0.095	-0.291	10,014	74.38	742	81.13	1192	84.31	310-311-311-311	79.35-79.10-79.42-78.78	[23]
*O. moubata*	P	NC_004357	14,398	72.26	0.067	-0.379	10,890	71.35	686	74.20	1212	76.90	342	71.64	[38]
*O. porcinus*	P	NC_005820	14,378	70.98	0.059	-0.355	10,877	70.11	691	74.38	1207	74.48	338	69.53	[27]
*R. sanguineus*	P	NC_002074	14,710	77.96	-0.034	-0.098	10,803	77.96	687	79.18	1190	81.34	303–305	67.33-66.56	[37]
*S. magnus*	A	NC_011574	13,818	74.59	-0.020	-0.037	10,560	74.44	609	74.38	992	74.38	1018	75.66	[22]
*T. urticae*	A	NC_010526	13,103	84.27	0.026	-0.016	10,226	84.00	646	85.91	991	85.27	44	95.45	[18]
*U. foilii*^g^	A	NC_011036	14,738	72.95	0.201	-0.279	10,679	71.83	649	74.35	1016	74.35	387–644	76.49–77.33	unpub.
*V. destructor*	P	NC_004454	16,477	80.02	-0.021	0.177	10,728	79.22	726	80.44	1149	83.12	2174	79.71	[25]
*W. hayashii*^g^	A	NC_010595	14,857	72.97	0.264	-0.305	10,573	73.01	625	75.05	1045	77.42	1403	68.28	unpub.
*Li. polyphemus*	^j^	NC_003057	14,985	67.60	0.111	-0.399	11,077	66.43	799	69.70	1296	71.00	348	81.3	[30,31]

* values were obtained from the corresponding GenBank flat-file in the NCBI database (status January 10, 2009)

^a ^D = *Dermatophagoides*, Am = *Amblyomma*, As = *Ascoschoengastia*, C = *Carios*, H = *Haemaphysalis*, I = *Ixodes*, Le = *Leptotrombidium*, M = *Metaseiulus*, O = *Ornithodoros*, R = *Rhipicephalus*, *S = Steganacarus*, T = *Tetranychus*, U = *Unionicola*, V = *Varroa*, W = *Walchia*, Li = *Limulus*

^b ^S = Acari superorder (A = Acariformes, P = Parasitiformes)

^c ^PCG = Protein coding genes

^d ^duplications of the control region were also considered

^e ^Ref = References; unpub = unpublished

^f ^GC- and AT-skew for the strand coding for *cox1*, calculated following [46]

^g ^for these species the largest non-coding region(s) was/were assumed to be the control region(s)

^h ^*L. pallidum *has a duplication of *16S-rRNA*; in this table the largest *16S-rRNA *gene is considered

^i ^only single copy genes were considered for protein coding gene length calculation of *M. occidentalis*

^j^*L. polyphemus *belongs to the order of the Xiphosura within the class of the Merostomata

All 37 genes present in a standard metazoan mt genome could be identified (Fig. 1). Gene overlap exists between *trnD/atp8 *(1 bp), *trnR*/*nad3 *(17 bp), *trnM*/*trnS*_2 _(12 bp), *trnP*/*trnV *(11 bp), *trnV*/*trnK *(7 bp), *trnW*/*trnY *(1 bp), *trnY*/*nad1 *(4 bp), *trnI*/*trnQ *(1 bp) and *trnL*_1_/*trnC *(5 bp). No overlap was found between protein coding genes. Small non-coding regions (> 20 bp) are present between *trnS*_2_/*trnA *(25 bp), *trnA*/*trnP *(20 bp) and *nad1*/*nad6 *(28 bp). A large non-coding region is positioned between *trnF *and *trnS*_1 _(286 bp). Twenty-five genes of the mt genome of *D. pteronyssinus *are transcribed on the majority strand (J-strand), whereas the others are oriented on the minority strand (N-strand).

**Figure 3:**
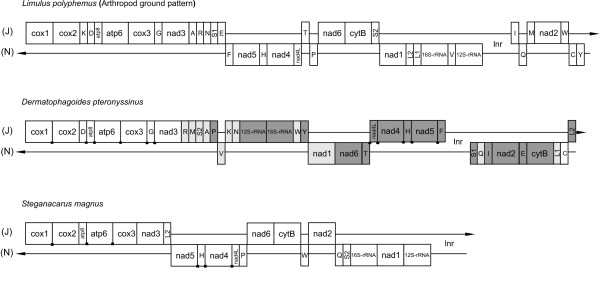
**Mitochondrial gene arrangement of Limulus polyphemus, Dermatophagoides pteronyssinus and Steganacarus magnus**. Graphical linearisation of mt genomes is presented according to [32]. Gene sizes are not drawn to scale. J stands for majority and N for minority strand. Protein coding and rRNA genes are abbreviated as in the abbreviations section. tRNA genes are abbreviated using the one-letter amino acid code, with L_1 _= CUN; L_2 _= UUR; S_1 _= AGN; S_2 _= UCN. White boxes represent genes with the same relative position as in the arthropod ground pattern, *L. polyphemus*. Light-gray boxes represent genes that changed positions relative to *L. polyphemus*; dark-gray boxes represent genes that changed both position and orientation. Circular dots between the genes of *D. pteronyssinus *represent conserved gene boundaries compared to *L. polyphemus*. Square dots between the genes of *S. magnus *represent conserved gene boundaries compared to *D. pteronyssinus*.

The mt genome of the horseshoe crab *Limulus polyphemus *is considered to represent the ground pattern for arthropod mt genomes [30,31]. Comparing the *D. pteronyssinus *genome to this sequence revealed that only 11 of the 38 gene boundaries in *L. polyphemus *are conserved in *D. pteronyssinus *(Fig. 3 [32]). Moreover, by making use of the pattern search function in the Mitome-database ([33], status January 10, 2009), the mt gene order of *D. pteronyssinus *appeared to be unique among arthropods. Remarkably, the relative position of *trnL*_2 _(between *nad1 *and *16S-rRNA*), which differentiates the Chelicerata, Myriapoda and Onychophora from the Insecta and Crustacea according to Boore [12,34], is not conserved. However, Boore's hypothesis was based on mt genome data from only 2 Chelicerata that were available in 1998. At present, 41 complete chelicerate mt genomes are available in the NCBI-database ([19], status January 10, 2009). Out of these, only 29 depict the specific arrangement of *trnL*_2 _between *nad1 *and *16S-rRNA *(see additional file 1 for an overview of gene arrangements of chelicerate mt genomes). This illustrates that care should be taken when general rules are deduced from limited datasets.

Mt gene arrangements have already provided strong support toward the resolution of several long-standing controversial phylogenetic relationships [12]. Surprisingly, the mt gene order of *D. pteronyssinus *differs considerably from that of other mites (see additional file 1). Comparing the *D. pteronyssinus *mt genome to the mt sequence of the oribatid *S. magnus *[22], the closest relative of *D. pteronyssinus*, revealed that only 6 of the 22 gene boundaries in *S. magnus *are conserved in *D. pteronyssinus *(Fig. 3).

Extending this analysis to the other Acari mt genomes showed that in several cases the set of neighboured genes that were not separated during the evolution [35] was greater between members of different superorders (e.g. *D. pteronyssinus *(Acariformes) and *Rhipicephalus sanguineus *(Parasitiformes)) than between members of the same superorder (e.g. *D. pteronyssinus *(Acariformes) and *T. urticae *(Acariformes)) (Table2). Exclusion of tRNAs in our analysis showed a similar trend, suggesting that protein coding genes were also involved in mt gene rearrangements. These results indicate that mt gene orders seem less useful for deduction of phylogenetic relationships between superorders within the Acari. However, comparing gene order might be more powerful to establish phylogenetic relations within families, as was previously proposed [14,36]. In the case of the Ixodidae family, it was shown that the division of Prostriata (*Ixodes *sp.) and Metastriata (*R. sanguineus*, *Amblyomma triguttatum*, *Haemaphysalis flava*) could be linked to mt gene arrangements [37,38].

**Table 2:** Pairwise common interval distance matrix of mt gene orders of Acari*.

	*Dp*	*I*	*Rs*	*Vd*	*La*	*Tu*	*A*	*Wh*	*Uf*	*Sm*
*D. pteronyssinus *(*Dp*)	**1326**/*204*	**70**	**74**	**46**	**18**	**14**	**18**	**18**	**16**	**-**
*Ixodes *sp. (*I*) ^a^	*56*	**1326**/*204*	**388**	**278**	**34**	**26**	**68**	**70**	**78**	**-**
*R. sanguineus *(*Rs*) ^b^	*86*	*78*	**1326**/*204*	**202**	**36**	**26**	**72**	**70**	**74**	**-**
*V. destructor *(*Vd*)	*56*	*204*	*78*	**1326**/*204*	**36**	**18**	**60**	**60**	**54**	**-**
*L. akamushi *(*La*) ^c^	*20*	*36*	*24*	*36*	**1326**/*204*	**20**	**100**	**98**	**90**	**-**
*T. urticae *(*Tu*)	*22*	*26*	*22*	*26*	*22*	**1326**/*204*	**20**	**20**	**16**	**-**
*Ascoschoengastia *sp.(*A*)	*34*	*38*	*46*	*38*	*44*	*28*	**1326**/*204*	**278**	**130**	**-**
*W. hayashii *(*Wh*)	*24*	*48*	*32*	*48*	*32*	*46*	*40*	**1326**/*204*	**122**	**-**
*U. foilii *(*Uf*)	*30*	*90*	*44*	*90*	*38*	*32*	*40*	*42*	**1326**/*204*	**-**
*S. magnus *(*Sm*) ^d^	*60*	*132*	*72*	*132*	*28*	*22*	*34*	*62*	*80*	-/*204*

*bold numbers represent pairwise common interval distances between mt gene orders of Acari (37 genes in total), while italic numbers represent pairwise common interval distances between mt gene orders of Acari without tRNAs (15 genes in total) (*L. pallidum *and *M. occidentalis *were excluded from the dataset as the genomes contain duplicated genes whereas other genes are absent). Accession numbers of Acari mt genomes are listed in Table 1.

^a ^similar gene order as *L. polyphemus*, *Ornithodoros *sp. and *C. capensis*

^b ^similar gene order as *H. flava *and *A. triguttatum*

^c ^similar gene order as *L. deliense*

^d ^due to lack of tRNAs only protein coding gene and rRNA order comparison was possible

### Base composition and codon usage

The overall AT-content of the mt genome of *D. pteronyssinus *is 72.6% (Table 1). This is within the range of the average AT-content of Acari mt genomes (74.6 +/- 4.0%). The high AT-content is reflected in the codon usage (Table 3 [39]) with nucleotides 'A' and 'T' preferred over 'C' and 'G' on the wobble position and the predominant use of codons deficient in 'C' or 'G'. For example, the most frequently used codons are TTT (F) (105 codons per 1000 codons) and TTA (L_2_) (78 codons per 1000 codons).

**Table 3:** Relative synonymous codon usage (RSCU) and number of codons per 1000 codons (NC1000) in the protein coding genes of the mitochondrial genome of *D. pteronyssinus*.

Amino acid	codon	RSCU*	NC1000	Amino acid	codon	RSCU	NC1000	Amino acid	codon	RSCU	NC1000	Amino acid	codon	RSCU	NC1000
**F**	TTC	0.24	14.14	**S2**	TCA	1.13	15.52	**Y**	TAC	0.48	11.09	**C**	TGC	0.33	2.77
	TTT	1.76	105.60		TCC	0.43	5.82		TAT	1.52	35.20		TGT	1.67	14.14
**L2**	TTA	3.43	78.71		TCG	0.14	1.94					**W**	TGA	1.11	12.75
	TTG	0.81	18.57		TCT	3.81	52.11						TGG	0.89	10.25
															
**L1**	CTA	0.74	16.91	**P**	CCA	1.53	14.41	**H**	CAC	0.50	4.16	**R**	CGA	1.37	3.88
	CTC	0.10	2.22		CCC	0.35	3.33		CAT	1.50	12.47		CGC	0.00	0.00
	CTG	0.10	2.22		CCG	0.24	2.22	**Q**	CAA	1.68	8.59		CGG	0.49	1.39
	CTT	0.83	19.12		CCT	1.88	17.74		CAG	0.32	1.66		CGT	2.15	6.10
															
**I**	ATC	0.40	16.08	**T**	ACA	1.33	13.58	**N**	AAC	0.63	10.53	**S1**	AGA	0.99	13.58
	ATT	1.60	64.30		ACC	0.44	4.43		AAT	1.37	23.00		AGC	0.16	2.22
**M**	ATA	1.58	57.65		ACG	0.05	0.55	**K**	AAA	1.67	28.55		AGG	0.61	8.31
	ATG	0.42	15.52		ACT	2.18	22.17		AAG	0.33	5.54		AGT	0.73	9.98
															
**V**	GTA	1.09	21.06	**A**	GCA	0.71	4.99	**D**	GAC	0.69	6.93	**G**	GGA	0.96	13.86
	GTC	0.17	3.33		GCC	0.59	4.16		GAT	1.31	13.03		GGC	0.15	2.22
	GTG	0.43	8.31		GCG	0.16	1.11	**E**	GAA	0.95	10.81		GGG	1.45	21.06
	GTT	2.31	44.62		GCT	2.53	17.74		GAG	1.05	11.92		GGT	1.44	20.79

* RSCU is the number of times a particular codon is observed relative to the number of times a codon would be observed in the absence of any codon usage bias [39].

Metazoan mt genomes usually present a clear strand bias in nucleotide composition [40]. This is probably due to asymmetric patterns of mutations during transcription and replication when one strand remains transiently in a single-stranded state, making it more vulnerable to DNA damage [41]. However, in the case of mtDNA-replication, this hypothesis is not without controversy [42-45]. The strand bias in nucleotide composition can be measured as GC- and AT-skews ((G%-C%)/(G%+C%) and (A%-T%)/(A%+T%), respectively) [46]. The overall GC- and AT-skews of the J-strand of the *D. pteronyssinus *mt genome are 0.194 and -0.199, respectively. These are the most extreme values encountered within mite mt genomes up till now (Table 1) and they are reversed compared to the usual strand biases of metazoan mtDNA (negative GC-skew and positive AT-skew for the J-strand). Moreover, a positive GC-skew for mite mt genomes seems to be rare since at present, it was only encountered in *Varroa destructor*. Although hypothetical, it could be the result of a strand swap of the control region [40]. This region contains all initiation sites for transcription [47] and an inversion of the control region is expected to produce a global reversal of asymmetric mutational constraints in the mtDNA, resulting with time in a complete reversal of strand compositional bias [40]. The asymmetrical directional mutation pressure is also reflected in the codon usage of genes oriented in opposite directions [48]. Whereas NNG and NNU codons are preferred over NNA and NNC codons on the J-strand, genes on the N-strand show the exact opposite trend (see additional file 2 for an across-strand (N and J) comparison of frequencies of codons ending with the same nucleotide).

### Protein coding genes

Nine proteins are encoded by genes on the J-strand (cox1, cox2, cox3, atp6, atp8, nad3, nad4, nad4L, nad5), while four are encoded by genes on the N-strand (nad1, nad6, nad2, cytB). The total length (10,826 bp) and AT-content (71.61%) of the protein-coding genes are within the range of values typical for Acari (10,639.0 +/- 272.0 bp; 74.0 +/- 4.0%, respectively) (Table 1). Compared to other mite mt proteins, cox1, cox2 and cytB are best conserved. On the other hand, atp8, nad6 and nad4L showed lowest similarity values (see additional file 3 for the average identity and similarity % of mt proteins of *D. pteronyssinus*).

Start and stop codons were determined based on alignments with the corresponding genes and proteins of other mite species. In the case of stop codons, we could also benefit from available expressed sequence tags (ESTs) of *D. pteronyssinus *(n = 1797) and *D. farinae *(n = 1735) (Fig. 1) [49]. As for other metazoan mt proteins, unorthodox initiation codons are used [20] (see additional file 4 for start and stop codons of protein coding genes of Acari mt genomes). Eight genes (*cox2, atp6, cox3, nad3, nad6, nad4L, nad4, cytB*) use the standard ATG start codon, 3 genes (*cox1, nad1, nad2*) start with ATA and *nad5 *initiates with ATT. *atp8 *most likely starts with codon TTG.

Eleven genes employ a complete translation termination codon, either TAG (*cox1, cox3*) or TAA (*cox2, atp8, atp6, nad1, nad3, nad6, nad4L, nad5, cytB*). With the exception of *nad3*, *atp8 *and *nad4L*, *D. pteronyssinus *ESTs for all these genes confirmed the position of the stop codon (Fig. 1, see additional file 5 for sequences of *Dermatophagoides *ESTs covering the mt genome of *D. pteronyssinus*). Berthier *et al. *[50] showed that the adjacent genes, *nad4L*/*nad4 *and *atp8*/*atp6*, were transcribed and translated as a bicistronic mRNA in the model organism *Drosophila melanogaster*. However, as no ESTs were found that aligned with the *nad4L/nad4 *and *atp8/atp6 *gene boundaries, it could not be confirmed whether this was also the case for *D. pteronyssinus*. Despite its efficiency, the use of sequence alignments to determine the position of stop codons resulted in several cases in overlapping genes. For example, based on a highly conserved tryptophan at the C-terminal end of Acari nad3 proteins, a stop codon was positioned despite the resulting 17 bp overlap with *trnaR*. The two remaining genes (*nad2 *and *nad4*) are likely equipped with a truncated stop codon (T). Polyadenylation of the mRNA is needed in these cases to form a fully functional TAA stop codon [51]. Although speculative, ESTs of *D. farinae *confirm the truncated stop of *nad4 *(Fig. 1, see additional file 5).

### Transfer RNAs

**Figure 4:**
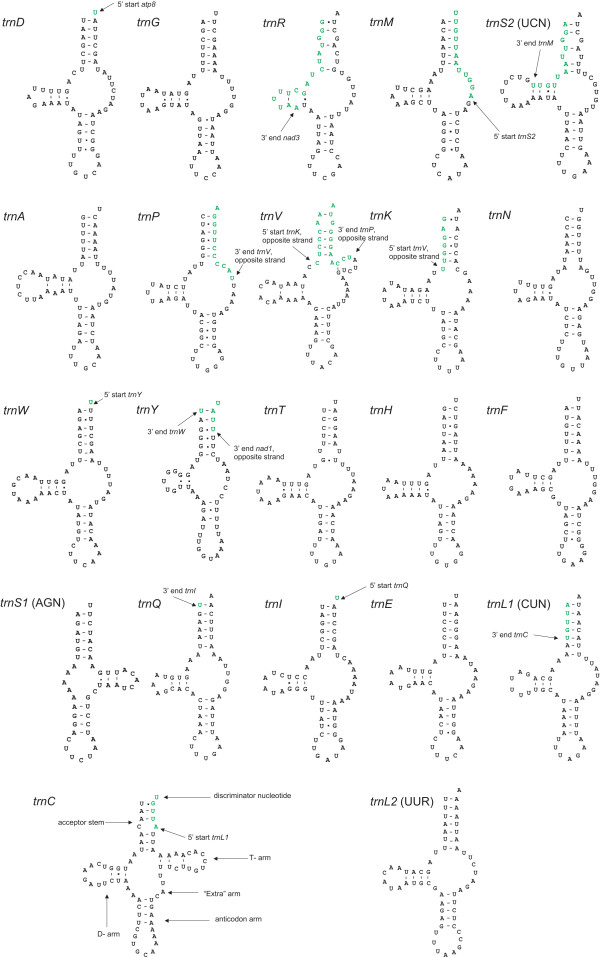
**Inferred secondary structures of the 22 mitochondrial tRNAs from *D. pteronyssinus***. tRNAs are shown in the order of occurrence in the mt genome starting from *cox1*. Locations of adjacent gene boundaries are indicated with arrows. Green font indicates that the sequence is part of the adjacent gene. Inferred Watson-Crick bonds are illustrated by lines, whereas GU bonds are illustrated by dots.

Fourteen tRNAs are encoded on the J-strand and 8 on the N-strand (Fig. 1). Secondary structures were predicted for all tRNAs (Fig. 4). With the exception of *trnS*_1 _(UCU instead of GCU in *L. pallidum*) and *trnP *(UGG instead of AGG in *S. magnus*), all anticodon sequences were identical to those of *L. pallidum *and *S. magnus*, the only acariform mites for which tRNA secondary structures have been reported [22,24]. Usually, T is in the first anticodon position for tRNAs that recognise either four-fold degenerate codon families or NNR codons. G is usually in this position only to specifically recognize NNY-codons [52]. Except for *trnM*, all of the *D. pteronyssinus *mt tRNAs follow this pattern. *trnM *has the anticodon CAT (to recognise both ATG and ATA), which is the case for almost all animal mt systems [52] (Fig. 4).

Only one tRNA lacks the D-arm: *trnS*_1_, as is common for most metazoans. With the exception of *trnC*, *trnV *and *trnS*_1_, all tRNAs have T-arm variable loops (TV replacement loops) instead of the T-arm. Similar structures were found for tRNAs of *L. pallidum *[24] and *S. magnus *[22]. The absence of the T-arm is a typical feature for tRNAs of Chelicerata belonging to the orders of the Araneae, Scorpiones and Thelyphonida. However, other taxa within the Chelicerata (Amblypygi, Opiliones, Ricinulei, Solifugae and ticks) possess typical metazoan cloverleaf tRNAs [13]. Masta and Boore [13] suggested a multi-step evolutionary process in an attempt to understand how so many tRNAs in these chelicerate groups could lose their T-arm. According to this speculative theory, changes in mt ribosomes, resulting in the fact that the loss of arms from tRNAs was tolerated [53,54], and/or changes in specific elongation factors [55-57] are considered as a first step in this process.

Only 7 of the 22 tRNAs have a completely matched 7 bp acceptor stem (*trnG, trnW, trnH, trnF, trnE*, *trnL*_1 _and *trnL*_2_). A maximum of 3 mismatches in this stem is found in *trnR*. In contrast, almost all tRNAs (18) possess a completely matched 5 bp anticodon stem. *trnC, trnS*_1 _and *trnN *have a single mismatch whereas *trnY *has two mismatches in this stem. All tRNAs, except *trnL*_2_, have a symmetric anticodon loop consisting of 2 bp up- and 2 bp downstream of the 3 bp anticodon. The anticodon loop of *trnL*_2 _consists of 2 nucleotides preceding the anticodon and 3 nucleotides immediately following it. This kind of aberrant anticodon loops have also been reported for the two-humped camel *Camelus bactrianus ferus *(*trnS*_2_) [58] and the scorpion *Mesobuthus gibbosus *(*trnH *and *trnN*) [59]. As mentioned before, sequences of some tRNAs overlap with neighbouring genes. The extreme examples are *trnR, trnS*_2 _and *trnV*. *trnR *overlaps with the adjacent gene *nad3 *on the same strand for 17 bp at its 3'-end whereas *trnS*_2 _overlaps with the adjacent gene *trnM *on the same strand for 12 bp at its 3'-end. *trnV *overlaps with the adjacent gene *trnP *on the opposite strand for 11 bp at its 3'-end and with *trnK *on the opposite strand for 7 bp at its 5'-start. Despite these overlaps, we consider these genes not likely to be pseudogenes. First of all, their sequence is relatively well conserved when compared to corresponding genes of other Acari. Secondly, besides sequence conservation they depict a conserved secondary structure. Thirdly, an EST [GenBank: CB284825] of the related species *D. farinae *was found corresponding to the region covering *trnR*, *trnM *and *trnS*_2 _of *D. pteronyssinus *indicating that the genes are expressed (see additional file 6 for an alignment of *trnR*, *trnM *and *trnS*_2 _of *D. pteronyssinus *with an EST of *D. farinae*). Finally, and most importantly, stem mismatches and sequence overlap are not uncommon for mt tRNAs of arachnids [13,60], and are probably repaired by a post-transcriptional editing process [54,61].

### Non-coding regions

The largest non-coding region (286 bp) is flanked by *trnF *and *trnS*_1_. It is highly enriched in AT (91.61%) and can form stable stem-loop secondary structures. Based on these features, it possibly functions as a control region [20,62]. With the exception of *T. urticae *(95.45%), it has the highest AT-content of all Acari mt control regions (Table 1). The position of the non-coding region differs from most insect and arachnid mt genomes, where the region is mostly located in close proximity to *12S-rRNA *([62], see additional file 1).

Based on the sequence pattern, the control region can be subdivided in a repeat region and a stem-loop region. The first region (11,491–11,528 bp) contains several AT-repeats. In order to verify the exact number of repeats we resequenced this region. For this purpose, two flanking primers, Dp-Ms-F and Dp-Ms-R, were synthesised spanning approximately 700 bp. The PCR product was cloned and ten independent clones were sequenced. This revealed that the number of AT-repeats varied between 7 to 28, suggesting that this domain can be considered as a microsatellite [63]. This is remarkable as a mt microsatellite was never reported before for species belonging to the Chelicerata. Also in metazoan mtDNA such microsatellites are rare and have, to our knowledge, only been reported for butterflies [64], a dragonfish, *Scleropages formosus *[65], a bat, *Myotis bechsteinii *[66], a turtle, *Pelomedusa subrufa *[67] and several seal species [68-70].

**Figure 5:**
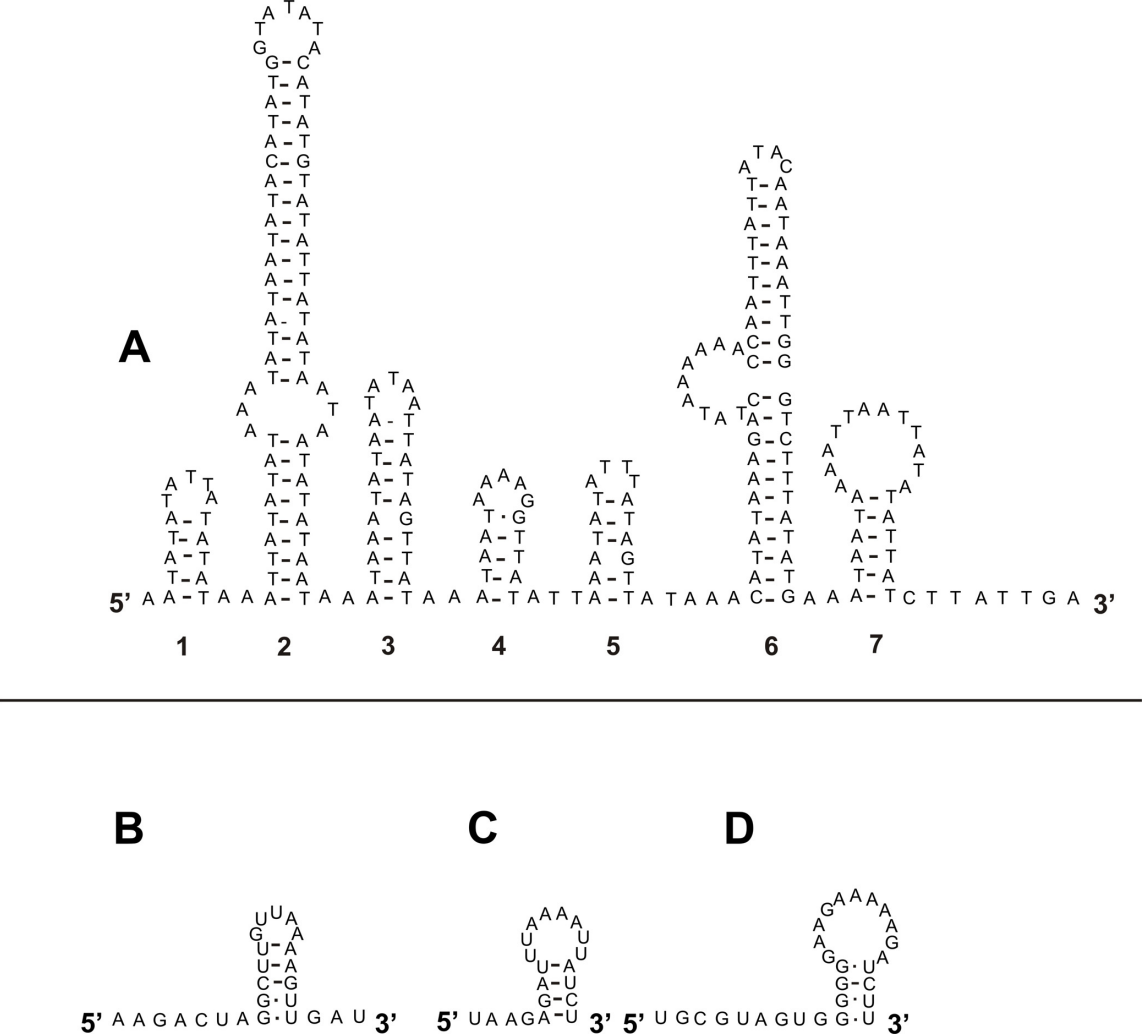
**Secondary structures of non-coding regions of the mt genome of *D. pteronyssinus***. Secondary structure of non-coding regions between (A) *trnF *and *trnS*_1 _(large non-coding region); (B) *trnS*_2 _and *trnA; *(C) *trnA *and *trnP; *(D) *nad1 *and *nad6*. All structures were constructed using Mfold [103]. Inferred Watson-Crick bonds are illustrated by lines, whereas GU bonds are illustrated by dots.

The second region (11,529–11,768 bp) holds two short palindromic sequences, TACAT and ATGTA, which are conserved in mt genomes of mammals [71] and fishes [65,72]. They can form a stable stem-loop structure (Fig. 5-A2), which might be involved as a recognition site for the arrest of J-strand synthesis [71]. Near this region other stem-loop structures could be folded (Fig. 5-A) but none of them had flanking sequences similar to those that are conserved in the control region of the mt genome of insects [62] and metastriate ticks [37].

As described before, four other stretches of non-coding nucleotides were found outside the control region. These short sequences can fold into stable stem-loop structures (Fig. 5-B, C, D) which may function as splicing recognition sites during processing of the transcripts [73].

### Ribosomal RNAs

*12S-rRNA *and *16S-rRNA *are located on the J-strand. This does not coincide with their position in most Chelicerata where they are located on the N-strand (see additional file 1). The AT-contents of both genes are comparable (72.9% and 76.1% for the *12S- *and *16S-RNA*, respectively) and are within the range of rRNAs of other Acari (76.5 +/- 4.2%; 78.0 +/- 4.1%, respectively). The sizes of the rRNAs (665 bp and 1078 bp) are slightly larger than those of other acariform mite rRNAs (626.0 +/- 29.9 bp and 1018.5 +/- 21.3 bp) but are shorter than those found in the Parasitiformes (706.0 +/- 17.5 bp and 1207.3 +/- 31.4 bp) (Table 1).

**Figure 6:**
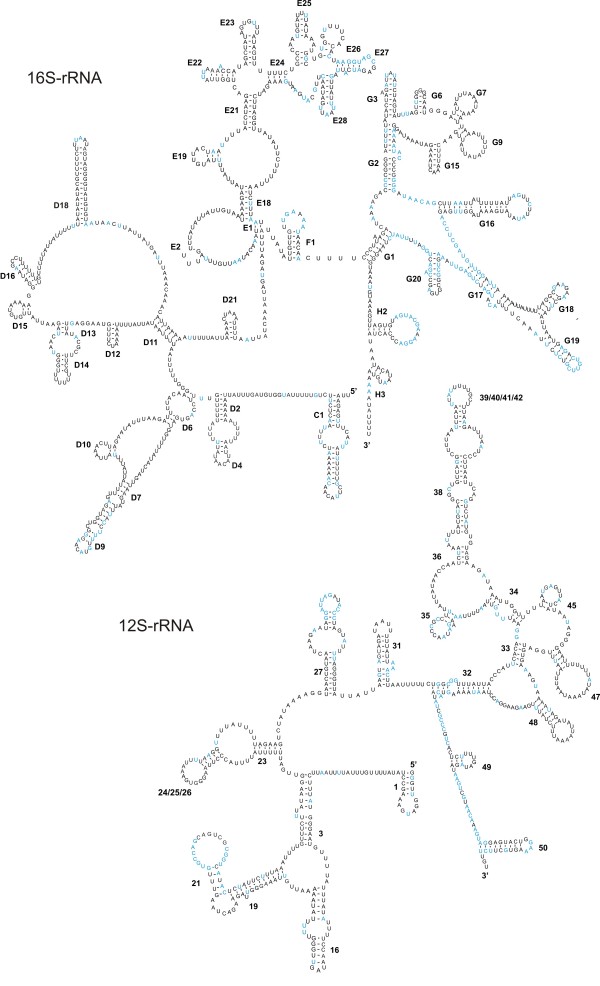
***16S-rRNA *and *12S-rRNA *secondary structures of the mitochondrial genome of *D. pteronyssinus***. The numbering of the stem-loops is after de Rijk *et al*. [75] for *16S-rRNA *and after van de Peer *et al*. [76] for *12S-rRNA*. Blue coloured nucleotides show 100% identity when aligned to *12S-rRNA *and *16S-rRNA *genes from other Acariformes (as listed in Table 1). Inferred Watson-Crick bonds are illustrated by lines, whereas GU bonds are illustrated by dots.

The *12S-rRNA *and *16S-rRNA *genes of *Leptotrombidium *species (Acariformes: Trombidiformes: Trombiculidae) are 23.4% and 23.5% shorter than their counterparts in *Drosophila yakuba*. This substantial reduction is mainly caused by the loss of stem-loop structures at the 5'-end of the rRNA genes [74]. To identify whether similar domains are absent in the rRNAs of *D. pteronyssinus*, we constructed their secondary structures (Fig. 6 [75,76]). This revealed that the *D. pteronyssinus 12S-rRNA *indeed lacks similar stem-loops as *L. pallidum*, compared to *D. yakuba*. The structure also revealed 1 additional stem-loop (stem-loop 1) not present in *12S-rRNA *of *L. pallidum*. Like in *L. pallidum*, one stem-loop replaces three stem-loops (24, 25 and 26) whereas another replaces a region of four stem-loops (39, 40, 41 and 42) of the *D. yakuba 12S-rRNA *[74]. Based on the modelled structure in combination with an alignment of other acariform *12S-rRNAs*, the greatest sequence conservation was found in the loop region of stem-loops 21 and 27 and the region between stem-loops 48 and 50.

In analogy to the *16S-rRNA *gene of *L. pallidum*, the main deletions of the *D. pteronyssinus 16S-rRNA *are located at the 5'-end. With the exception of D19, all stem-loops of *L. pallidum *are present in *D. pteronyssinus*. We also discovered three additional stem-loops (C1, E2 and E19) which are absent in the *16S-rRNA *of *L. pallidum*. The 3'-end of the *16S-rRNA *structure is best conserved compared to other acariform *16S-rRNAs*. This is in agreement with the idea that this region is the main component of the peptidyl-transferase centre, and as such most vulnerable to mutations [73]. Recently, the *12S-rRNA *and *16S-rRNA *secondary structures of *S. magnus *have been published [22]. The *12S-rRNA *structure of *S. magnus *has 5 extra stem-loops (2, 4, 5, 40 and 42) compared to the one of *D. pteronyssinus *whereas the *16S-rRNA *lacks 6 stem-loops (D4, D16, E1, E2, E19 and G9) and has 5 stem-loops (cd1, D1, D17, D19, G13) not present in the *16S-rRNA *of *D. pteronyssinus*.

It is still an open question how relatively well-conserved structures such as rRNAs can dramatically decrease in size while remaining functional. Wolstenholme *et al. *[53] and Masta [54] suggested a correlation between the occurrence of truncated rRNAs (compared to *Drosophila*) and the loss of the T-arm in tRNAs. The coincidence of short rRNAs and missing T-arms in tRNAs was also observed in *S. magnus, L. pallidum *and *D. pteronyssinus*. Other acariform mites like *T. urticae*, *Ascoschoengastia *sp. and *Walchia hayashii *also exhibit short rRNAs (Table 1) and the prediction of their tRNA secondary structures could further support this hypothesis. However, examples contradicting this hypothesis also occur e.g. pulmonate gastropods with tRNAs lacking T-arms have no truncated rRNAs. Therefore, it remains possible that truncation of both tRNAs and rRNA genes only reflects an independent trend towards minimisation of the mt genome as suggested by Yamazaki *et al*. [77].

### Phylogenetic analysis

A phylogenetic tree was constructed based on nucleotide and amino acid sequences from all mt protein coding genes of Acari. The ILD-test [78] indicated a significant incongruence (P = 0.01) among data set partitions for nucleotide alignments and low congruence (P = 0.07) among data set partitions for amino acid alignments. A considerable debate exists on the utility of this test [79-84]. However, the principle of Kluge [85] implies that all data should always be included in a combined analysis for any phylogenetic problem and therefore we combined data partitions for both amino acid and nucleotide alignments for phylogenetic analysis. A maximum parsimony (MP) analysis based on nucleotide alignments (data not shown) grouped *V. destructor *(Parasitiformes) within the Acariformes, close to *D. pteronyssinus*. This is in contrast with the generally accepted view on the phylogeny of the Acariformes and Parasitiformes [2,3]. As mentioned before, *V. destructor *and *D. pteronyssinus *both have a reversal of asymmetrical mutation pattern. When such reversals occurred independently, *D. pteronyssinus *and *V. destructor *could have acquired a similar base composition and as a consequence group together due to the long-branch attraction (LBA) phenomenon [40,86]. Model-based methods such as maximum likelihood (ML) and Bayesian inference (BI) are less sensitive to LBA [40,87] and were for this reason considered for phylogenetic analysis.

**Figure 7:**
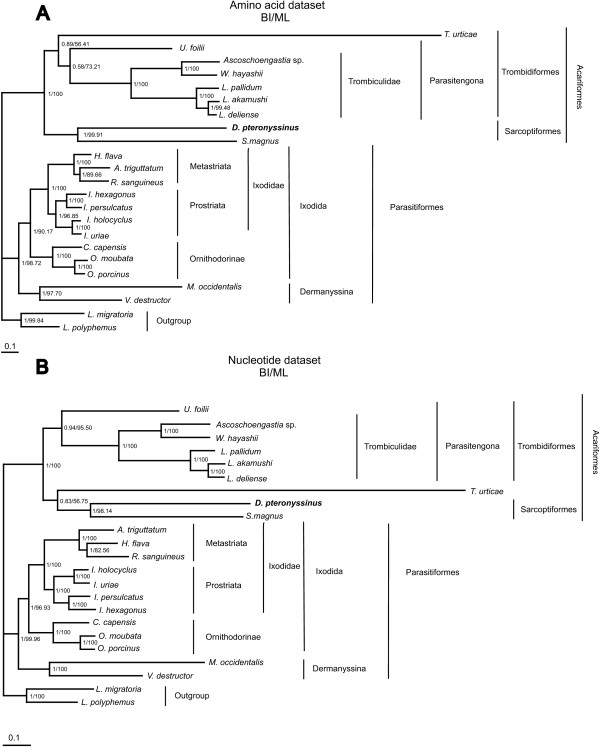
**Phylogenetic trees of Acari relationships**. Trees were inferred from amino acid (A) and nucleotide (B) datasets. All protein coding gene sequences were aligned and concatenated; ambiguously aligned regions were omitted by Gblocks 0.91b [105]. Trees were rooted with two outgroup taxa (*L. polyphemus *and *L. migratoria*). Numbers behind the branching points are percentages from Bayesian posterior probabilities (left) and ML bootstrapping (right). Accession numbers for the different Acari mt genomes are listed in Table 1.

ML and BI analysis performed on the amino acid data set resolved trees with an identical topology (Fig. 7-A) in which *D. pteronyssinus *clusters with *S. magnus*, forming a sistergroup of the Trombidiformes. This is in agreement with the most recent views on the classification of the Acariformes [2-4]. The nucleotide data set resulted in similar trees, confirming the evolutionary position of *D. pteronyssinus *(Fig. 7-B). The only major inconsistency over the trees was the position of *T. urticae*. Although this species is generally considered as a member of the Trombidiformes [2-4], it was clustered with the sarcoptiform mites *D. pteronyssinus *and *S. magnus *in the trees based on the nucleotide dataset. (Fig. 7-B). However, the position in the different trees is questionable as it is supported by low bootstrap values/Bayesian posterior probabilities (Fig. 7-A/B). Adding additional mt genome data from closely related taxa of *T. urticae *and from taxa located between *T. urticae *and Trombiculidae would probably position *T. urticae *with higher support values within the Trombidiformes.

In the trees based on the nucleotide dataset, *H. flava *is, compared to *A. triguttatum*, evolutionary closer related to *R. sanguineus *while in the trees based on the amino acid dataset this is the opposite. However, as the clustering of *H. flava *and *R. sanguineus *is in agreement with the most recent views on the classification of the Ixodida [88,89], we consider the nucleotide topology as the most correct one. Murrell *et al. *[90] considers the Parasitiformes to be paraphyletic with respect to the Opilioacariformes, but as there are no complete mt genomes of Opilioacariformes available, we were not able to verify this hypothesis.

## Conclusion

This is the first description of a complete mt genome of a species belonging to the Astigmata, a cohort within the Sarcoptiformes. Although the length, gene and AT-content are similar to other Acari mtDNA, the mt genome of *D. pteronyssinus *exhibits some interesting features. The gene order of *D. pteronyssinus *is completely different from that of other Acari mt genomes. Gene order comparison indicated that mt gene orders seem less useful for deduction of phylogenetic relationships between superorders within the Acari. GC- and AT-skews of the J-strand were very large and reversed as compared to those found in most metazoan mtDNA.

Compared to parasitiform mites, both *D. pteronyssinus *rRNAs were considerably shorter and almost all transfer RNAs lacked the T-arm. It would be interesting to investigate whether the occurrence of truncated rRNAs and the loss of the T-arm in tRNAs are correlated or just a trend toward minimisation of the mt genome. Finally, phylogenetic analysis using concatenated mt gene sequences succeeded in recovering Acari relationships concordant with traditional views of phylogeny of Acari.

## Methods

### Mite identification

Upon arrival in the laboratory, mites were identified as *D. pteronyssinus *by J. Witters (ILVO, Belgium) and F. Th. M. Spieksma (Laboratory of Aerobiology, LUMC, The Netherlands) using morphological characteristics. To back up this identification, molecular techniques were applied. For this purpose DNA was extracted and used as a template for PCR. Primers 12SID-F and 12SID-R (see additional file 7 for primer sequences) successfully amplified a 316 bp fragment. BLASTn searches against non-redundant nucleotide sequences using the amplified fragment as query resulted in a perfect match with a mt *12S-rRNA *sequence of *D. pteronyssinus *[GenBank: AF529911].

### Mite strain, mass rearing and isolation

The initial *D. pteronyssinus *culture was provided by D. Bylemans (Janssen Pharmaceutica, Belgium). Mites were cultured on a 1:1 mixture of Premium Gold (Vitacraft, Germany) and beard shavings at 75% R.H., 25°C and permanent dark conditions [91,92]. Mites were isolated from the colony using a modified heat-escape technique [93,94]. Briefly, mite cultures were transferred to small plastic petri dishes (75 mm in diameter, 28 mm high) with a lid on top. These dishes were placed in the dark on a hot plate set at 45°C (Bekso, Belgium). After 15–20 minutes the mites moved away from the heat source, formed groups on the lid of the petri dish and could be collected using a fine hair brush.

### DNA extraction

Approximately 1000 *D. pteronyssinus *mites were collected in an Eppendorf tube and were ground in 800 μl SDS-lysis buffer (400 mM NaCl, 200 mM TRIS, 10 mM EDTA, 2% SDS) using a small sterile plastic pestle (Eppendorf, Germany). After incubation for 30 min at 60°C under continuous rotation, a standard phenol-chloroform extraction was performed [95]. Total genomic DNA was precipitated with 0.7 volumes of isopropanol at 4°C for 1 hour, centrifuged for 45 minutes at 21,000 × g and washed with 70% ethanol. Precipitated DNA was resolved in 50 μl 0.1 M Tris pH 8.2.

### PCR

Standard PCR (amplicon < 500 bp) was performed in 50 μl volumes (38.5 μl double-distilled water; 5 μl buffer; 2 mM MgCl_2_; 0.2 mM dNTP-mix; 0.2 μM of each primer; 1 μl template DNA and 0.5 μl *Taq *polymerase (Invitrogen, Belgium). PCR conditions were as follows: 2' 94°C, 35 × (20" 92°C, 30" 53°C, 1' 72°C) and 2' 72°C. The annealing time was extended to 1 minute and the primer concentration was increased to 2 μM when degenerate primers were used. Long PCR (amplicon > 500 bp) was performed with the Expand Long Range Kit (Roche, Switzerland) in 50 μl volumes (28.5 μl double-distilled water; 10 μl buffer; 0.5 mM dNTP-mix; 0.3 μM of each primer; 4 μl 100% DMSO; 1 μl template DNA and 1 μl enzyme-mix). PCR conditions were: 2' 94°C, 10 × (10" 92°C, 20" at a temperature that varies depending on the primers, 1'/kb 58°C), 25 × (10" 92°C, 20" at a temperature that varies depending on the primers, 1'/kb 58°C with 20" added for every consecutive cycle) and 7' 58°C. All PCR products were separated by electrophoresis on a 1% agarose gel and visualised by EtBr staining. Fragments (amplicon < 1000 bp) of interest were excised from gel, purified with the QIAquick PCR Purification Kit (Qiagen, Belgium) and cloned into the pGEM-T vector (Promega, Belgium). After heat-shock transformation of *E. coli *(DH5α) cells, plasmid DNA was obtained by miniprep and inserted fragments were sequenced with SP6 and T7-primers. Long PCR products were sequenced by primer-walking. All sequencing reactions were performed by AGOWA sequencing service.

### Amplification of the mt genome

Primers COXI-F and 12S-R, based on partial *D. pteronyssinus cox1 *and *12S-rRNA *sequences [GenBank: AY525570 and AF529911, respectively] (see additional file 7 for primer sequences), successfully amplified a 4.6 kb sequence of the mt genome of *D. pteronyssinus*. Degenerate primers CYTB-F-Deg and CYTB-R-Deg (see additional file 7 for primer sequences), designed on conserved regions of Acari *cytB*, amplified a partial *cytB *sequence from *D. pteronyssinus*. A specific primer COXI-R, designed from the 3' end of the 4.5 kb sequence in combination with the primer CYTB-F, designed from the partial *cytB *sequence, successfully amplified a 2.2 kb sequence. Another primer CYTB-R, designed from the 5'-end of this 2.2 kb sequence, in combination with the primer 12S-F successfully amplified a 8.6 kb sequence, making the mt genome sequence complete.

### Annotation and bioinformatics analysis

The complete genomic sequence was assembled and annotated using VectorNTI (Invitrogen, Belgium) according to Masta and Boore [60]. Open reading frames (ORFs) were identified with the program Getorf from the EMBOSS-package [96]. The obtained ORFs were used as query in BLASTp [97] searches against the non-redundant protein database at NCBI. Two large non-protein-coding regions were candidates for the rRNAs (16S and 12S respectively). The boundaries were identified based on alignments and secondary structures of rRNA genes of other mite species. Sixteen of the 22 tRNAs were identified by tRNA-scan SE [98] with a cove cutoff score of 0.1 and the tRNA-model set to "nematode mito". The remaining tRNAs (*trnM*, *trnV*, *trnY*, *trnS*_1_, *trnI*, *trnC*) were determined in the unannotated regions by sequence similarity to tRNAs of other mite species. In order to obtain additional information on mt gene boundaries, BLASTn [97] searches of *D. pteronyssinus *tRNA, rRNA and protein encoding nucleotide sequences were carried out against ESTs [49] restricted to *Dermatophagoides *sequences (n = 3532). ESTs with statistically significant matches (E-value cutoff: 0.1) were collected, checked for vector contamination and aligned by Clustal W [99] as implemented in BioEdit 7.0.1 [100] against the appropriate nucleotide sequence of *D. pteronyssinus*. MatGAT 2.02 was used to calculate similarity and identity values [101] of mt proteins. The identification of gene subsets that appear consecutively in different genomes was performed by common interval distance analysis using CREx [102] (see additional file 8 for input data of the CREx program).

### Construction of secondary structures of RNAs and non-coding regions

Secondary structures of tRNAs were determined following the method of Masta and Boore [60]. Secondary structures of tRNAs were drawn with CorelDraw 12.0 (Corel Corporation, Canada). The rRNA genes of *D. pteronyssinus *were aligned with those of other Acariformes and conserved areas were identified. These regions were mapped on the published structures of *L. pallidum *rRNA [74]. Regions lacking significant homology were folded using Mfold [103]. Secondary structures of rRNAs were drawn using the RnaViz2 program [104] and afterwards modified with CorelDraw 12.0 (Corel Corporation, Canada). Secondary structures of non-coding regions were folded using Mfold [103]. When multiple secondary structures were possible, the most stable (lowest free energy (-ΔG)) one was preferred. Drawing and editing of these structures was done in a similar way as for rRNA secondary structures.

### Rolling circle amplification and restriction enzyme digestion

Extraction and rolling circle amplification of the mtDNA of *D. pteronyssinus *was done according to Van Leeuwen *et al. *[18]. Rolling circle amplified mtDNA was digested with two enzymes (*Xmn*I and *Eco*RI; New England Biolabs) following the manufacturer's instructions. Restriction digests were fractionated by agarose gel electrophoresis as described before.

### Phylogenetic analysis

Sequence data were obtained from 21 Acari species (for GenBank accession numbers see Table 1) and two outgroup taxa (*Limulus polyphemus *[GenBank: NC_003057] and *Locusta migratoria *[Genbank: NC_001712]). Only mite species with a completely sequenced mt genome were selected. Alignments from all mt protein-coding genes were used in phylogenetic analysis. Amino acid sequences and nucleotide sequences were aligned by Clustal W [99] as implemented in BioEdit 7.0.1 [100]. The nucleotide alignment was generated based on the protein alignment using codon alignment. Ambiguously aligned parts were omitted from the analysis by making use of Gblocks 0.91b [105], with default block parameters except for changing "allowed gap positions" to "with half". Abascal *et al. *[106] recently presented evidence that some insects and ticks use a modified mitochondrial code, with AGG coding for lysine rather than serine as in the standard invertebrate mitochondrial code. As 10 out of 20 Acari species in our dataset are ticks all positions aligning to AGG codons in the final amino acid alignment were removed.

For the nucleotide alignments the "codons" option was used in Gblocks 0.91b [105]. Due to the results of a saturation analysis [107] on single codon positions, implemented in DAMBE 4.2.13 [108], third codon positions were eliminated from the nucleotide alignment. An incongruence length difference test (ILD-test) [78] as implemented in PAUP* (version 4.0b10; [109]) was used to assess congruence among gene partitions.

Model selection was done with ProtTest 1.4 [110] for amino acid sequences and with Modeltest 3.7 [111] for nucleotide sequences. According to the Akaike information criterion, the mtART+G+I+F model was optimum for phylogenetic analysis with amino acid alignments and the GTR+I+G model was optimal for analysis with nucleotide alignments.

Two different analyses were performed. (1) Maximum likelihood (ML) analysis was performed using Treefinder [112], bootstrapping with 1000 pseudoreplicates (2) Bayesian inference (BI) was done with MrBayes 3.1.2 [113]. As the mtART model is not implemented in the current version of MrBayes, the mtREV+G+I model was used for phylogenetic analysis with the amino acid alignment. Four chains ran for 1,000,000 generations, while tree sampling was done every 100 generations. Burnin was calculated when the average standard deviation of split frequencies had declined to < 0.01. The remaining trees were used to calculate Bayesian posterior probabilities (BPP).

## Abbreviations

*12S-rRNA*: small (12S) rRNA subunit (gene); *16S-rRNA*: large (16S) rRNA subunit (gene); A: adenine; *atp6 *and *8*: ATPase subunit 6 and 8; BI: Bayesian inference; bp: base pairs; *cox1*-*3*: cytochrome oxidase subunits I-III; *cytB*: cytochrome b; C: cytosine; D-arm: dihydrouridine-arm of a tRNA secondary structure; EST: expressed sequence tag; G: guanine; J-strand: majority strand; lnr: large non-coding region/control region; MP: maximum parsimony; ML: maximum likelihood; mRNA: messenger RNA; mt: mitochondrial; N-strand: minority strand; *nad1*-*6 *and *nad4L*: NADH dehydrogenase subunits 1–6 and 4L; ORF: open reading frame; PCR: polymerase chain reaction; R.H.: relative humidity; rRNA: ribosomal RNA; T: thymine; T-arm: TΨC-arm of a tRNA secondary structure; *trnX *(where *X *is replaced by a one letter amino acid code for the corresponding amino acid, with L_1 _= CUN; L_2 _= UUR; S_1 _= AGN; S_2 _= UCN), transfer RNA.

## Authors' contributions

WD and TVL designed and conducted the experiments. WD and BV analysed data. WD wrote the manuscript. All authors read and approved the final manuscript.

## Supplementary Material

Additional file 1 **Mitochondrial genome arrangements of 42 Chelicerata**. Graphical linearisation of the mt genomes was done according to [32] (see Fig. 3). Corresponding GenBank accession numbers are between brackets. The position of *trnL*_2 _is grey shaded. Protein coding and rRNA genes are abbreviated as in the Abbreviations section; tRNA genes are abbreviated using the one-letter amino acid code. Small non-coding regions (> 50bp) are indicated as gaps between genes. Braces accentuate the duplicated region in the mt genome of *M. occidentalis*. Click here for file

Additional file 2 **Across-strand (N and J) comparison of frequencies of codons ending with the same nucleotide**. Values on the y-axis represent the sum of Relative Synonymous Codon Usage (RSCU) values (Table 3) of codons ending with the same nucleotide across all codon families (x-axis). Click here for file

Additional file 3 **Average identity and similarity % of mt proteins of *D. pteronyssinus***. For each protein of *D. pteronyssinus*, a similarity and identity value was calculated with the corresponding protein of other Acari species (as listed in Table 1), using pairwise global alignment. The obtained values were used to calculate an average identity and similarity % for each protein. Click here for file

**Additional file 4** **Start and stop codons of mt protein coding genes of complete mt genomes of Acari.** Click here for file

Additional file 5 **GenBank accession numbers and sequences of ESTs of *D. pteronyssinus *and *D. farinae *covering the mt genome of *****D. pteronyssinus***. Click here for file

Additional file 6 **Alignment of a mt genome fragment containing *trnaM*, *trnaR *and *trnaS_2 _*of *D. pteronyssinus *and an EST [GenBank: **CB284825] **of *D. farinae***. Anticodons and anticodon stems are red and green respectively. Acceptor-stems of *trnR, trnM *and *trnS*_2 _are underlined. The stop codon of *nad3 *is grey shaded. Click here for file

Additional file 7 **Primers and their sequences used to characterise the *D. pteronyssinus *mt genome.** Click here for file

Additional file 8 **Input data for CREx.** Click here for file

## References

[B1] Harvey MS (2002). The neglected cousins: What do we know about the smaller Arachnid orders?. J Arachnol.

[B2] Acari. The Mites. http://tolweb.org/Acari/2554/1996.12.13.

[B3] Dunlop JA, Alberti G (2008). The affinities of mites and ticks: a review. J Zool Syst Evol Res.

[B4] O'Connor BM, Griffiths DA, Bowman CE (1984). Phylogenetic relationships among higher taxa in the Acariformes, with particular reference to the Astigmata. Acarology VI.

[B5] Spieksma FTM (1997). Domestic mites from an acarologic perspectives. Allergy.

[B6] Voorhorst R, Spieksma FT, Varekamp H, Leupen MJ, Lyklema AW (1967). House-dust mite (*Dermatophagoides pteronyssinus*) and allergens it produces. Identity with house-dust allergen. J Allergy.

[B7] van Bronswijk JEMA (1981). House dust biology.

[B8] Arlian LG, Platts-Mills TAE (2001). The biology of dust mites and the remediation of mite allergens in allergic disease. J Allergy Clin Immunol.

[B9] Janson C, Anto J, Burney P, Chinn S, de Marco R, Heinrich J, Jarvis D, Kuenzli N, Leynaert B, Luczynska C, Neukirch F, Svanes C, Sunyer J, Wjst M (2001). The European Community Respiratory Health Survey: what are the main results so far?. Eur Respir J.

[B10] Dowton M, Castro LR, Austin AD (2002). Mitochondrial gene rearrangements as phylogenetic characters in the invertebrates: the examination of genome 'morphology'. Invertebr Syst.

[B11] Boore JL, Macey JR, Medina M (2005). Sequencing and comparing whole mitochondrial genomes of animals. Molecular Evolution: Producing the Biochemical Data, Part B.

[B12] Boore JL (2006). The use of genome-level characters for phylogenetic reconstruction. Trends Ecol Evol.

[B13] Masta SE, Boore JL (2008). Parallel evolution of truncated transfer RNA genes in arachnid mitochondrial genomes. Mol Biol Evol.

[B14] Curole JP, Kocher TD (1999). Mitogenomics: digging deeper with complete mitochondrial genomes. Trends Ecol Evol.

[B15] Cameron SL, Miller KB, D'Haese CA, Whiting MF, Barker SC (2004). Mitochondrial genome data alone are not enough to unambiguously resolve the relationships of Entognatha, Insecta and Crustacea sensu lato (Arthropoda). Cladistics.

[B16] Castro LR, Dowton M (2007). Mitochondrial genomes in the Hymenoptera and their utility as phylogenetic markers. Syst Entomol.

[B17] Dekeyser MA (2005). Acaricide mode of action. Pest Manag Sci.

[B18] Van Leeuwen T, Vanholme B, Van Pottelberge S, Van Nieuwenhuyse P, Nauen R, Tirry L, Denholm I (2008). Mitochondrial heteroplasmy and the evolution of insecticide resistance: Non-Mendelian inheritance in action. Proc Natl Acad Sci USA.

[B19] NCBI – National Center For Biotechnology Information. http://www.ncbi.nlm.nih.gov/.

[B20] Wolstenholme DR (1992). Animal mitochondrial DNA: structure and evolution. Int Rev Cytol.

[B21] Boore JL (1999). Animal mitochondrial genomes. Nucleic Acids Res.

[B22] Domes K, Maraun M, Scheu S, Cameron SL (2008). The complete mitochondrial genome of the sexual oribatid mite *Steganacarus magnus*: genome rearrangements and loss of tRNAs. BMC Genomics.

[B23] Jeyaprakash A, Hoy MA (2007). The mitochondrial genome of the predatory mite *Metaseiulus occidentalis *(Arthropoda: Chelicerata: Acari: Phytoseiidae) is unexpectedly large and contains several novel features. Gene.

[B24] Shao RF, Mitani H, Barker SC, Takahashi M, Fukunaga M (2005). Novel mitochondrial gene content and gene arrangement indicate illegitimate inter-mtDNA recombination in the chigger mite, *Leptotrombidium pallidum*. J Mol Evol.

[B25] Navajas M, Le Conte Y, Solignac M, Cros-Arteil S, Cornuet JM (2002). The complete sequence of the mitochondrial genome of the honeybee ectoparasite mite *Varroa destructor *(Acari: Mesostigmata). Mol Biol Evol.

[B26] Shao RF, Barker SC, Mitani H, Aoki Y, Fukunaga M (2005). Evolution of duplicate control regions in the mitochondrial genomes of metazoa: A case study with Australasian Ixodes ticks. Mol Biol Evol.

[B27] Mitani H, Talbert A, Fukunaga M (2004). New World relapsing fever *Borrelia *found in *Ornithodoros porcinus *ticks in central Tanzania. Microbiol Immunol.

[B28] Gorrochotegui-Escalante N, Black WC (2003). Amplifying whole insect genomes with multiple displacement amplification. Insect Mol Biol.

[B29] Howell N (1985). Anomalous electrophoretic mobility of mouse mtDNA restriction fragments. Plasmid.

[B30] Staton JL, Daehler LL, Brown WM (1997). Mitochondrial gene arrangement of the horseshoe crab *Limulus polyphemus *L: Conservation of major features among arthropod classes. Mol Biol Evol.

[B31] Lavrov DV, Boore JL, Brown WM (2000). The complete mitochondrial DNA sequence of the horseshoe crab *Limulus polyphemus*. Mol Biol Evol.

[B32] Fahrein K, Talarico G, Braband A, Podsiadlowski L (2007). The complete mitochondrial genome of *Pseudocellus pearsei *(Chelicerata: Ricinulei) and a comparison of mitochondrial gene rearrangements in Arachnida. BMC Genomics.

[B33] Lee YS, Oh J, Kim YU, Kim N, Yang S, Hwang UW (2008). Mitome: dynamic and interactive database for comparative mitochondrial genomics in metazoan animals. Nucleic Acids Res.

[B34] Boore JL, Lavrov DV, Brown WM (1998). Gene translocation links insects and crustaceans. Nature.

[B35] Berard S, Bergeron A, Chauve C, Paul C (2007). Perfect sorting by reversals is not always difficult. IEEE/ACM Trans Comput Biol Bioinform.

[B36] Nardi F, Carapelli A, Fanciulli PP, Dallai R, Frati F (2001). The complete mitochondrial DNA sequence of the basal Hexapod *Tetrodontophora bielanensis*: Evidence for heteroplasmy and tRNA translocations. Mol Biol Evol.

[B37] Black WC, Roehrdanz RL (1998). Mitochondrial gene order is not conserved in arthropods: Prostriate and metastriate tick mitochondrial genomes. Mol Biol Evol.

[B38] Shao R, Aoki Y, Mitani H, Tabuchi N, Barker SC, Fukunaga M (2004). The mitochondrial genomes of soft ticks have an arrangement of genes that has remained unchanged for over 400 million years. Insect Mol Biol.

[B39] Sharp PM, Cowe E, Higgins DG, Shields DC, Wolfe KH, Wright F (1988). Codon usage patterns in *Escherichia coli*, *Bacillus subtilis*, *Saccharomyces cerevisiae*, *Schizosaccharomyces pombe*, *Drosophila melanogaster *and *Homo sapiens *– A review on the considerable within species diversity. Nucleic Acids Res.

[B40] Hassanin A, Leger N, Deutsch J (2005). Evidence for multiple reversals of asymmetric mutational constraints during the evolution of the mitochondrial genome of Metazoa, and consequences for phylogenetic inferences. Syst Biol.

[B41] Francino MP, Ochman H (1997). Strand asymmetries in DNA evolution. Trends Genet.

[B42] Clayton DA (1991). Replication and transcription of vertebrate mitochondrial DNA. Annu Rev Cell Biol.

[B43] Yang MY, Bowmaker M, Reyes A, Vergani L, Angeli P, Gringeri E, Jacobs HT, Holt IJ (2002). Biased incorporation of ribonucleotides on the mitochondrial L-strand accounts for apparent strand-asymmetric DNA replication. Cell.

[B44] Yasukawa T, Yang MY, Jacobs HT, Holt IJ (2005). A bidirectional origin of replication maps to the major noncoding region of human mitochondrial DNA. Mol Cell.

[B45] Brown TA, Cecconi C, Tkachuk AN, Bustamante C, Clayton DA (2005). Replication of mitochondrial DNA occurs by strand displacement with alternative light-strand origins, not via a strand-coupled mechanism. Genes Dev.

[B46] Perna NT, Kocher TD (1995). Patterns of nucleotide composition at fourfold degenerate sites of animal mitochondrial genomes. J Mol Evol.

[B47] Taanman JW (1999). The mitochondrial genome: structure, transcription, translation and replication. Biochim Biophys Acta, Bioenerg.

[B48] Carapelli A, Comandi S, Convey P, Nardi F, Frati F (2008). The complete mitochondrial genome of the antartic springtail *Cryptopygus antarcticus *(Hexapoda: Collembola). BMC Genomics.

[B49] Gissi C, Pesole G (2003). Transcript mapping and genome annotation of ascidian mtDNA using EST data. Genome Res.

[B50] Berthier F, Renaud M, Alziari S, Durand R (1986). RNA mapping on Drosophila mitochondrial DNA – precursors and template strands. Nucleic Acids Res.

[B51] Ojala D, Montoya J, Attardi G (1981). Transfer-RNA punctuation model of RNA processing in human mitochondria. Nature.

[B52] Boore JL (2006). The complete sequence of the mitochondrial genome of *Nautilus macromphalus *(Mollusca: Cephalopoda). BMC Genomics.

[B53] Wolstenholme DR, Macfarlane JL, Okimoto R, Clary DO, Wahleithner JA (1987). Bizarre transfer-RNAs inferred from DNA-sequences of mitochondrial genomes of nematode worms. Proc Natl Acad Sci USA.

[B54] Masta SE (2000). Mitochondrial sequence evolution in spiders: Intraspecific variation in tRNAs lacking the T psi C arm. Mol Biol Evol.

[B55] Ohtsuki T, Watanabe Y, Takemoto C, Kawai G, Ueda T, Kita K, Kojima S, Kaziro Y, Nyborg J, Watanabe K (2001). An "elongated" translation elongation factor Tu for truncated tRNAs in nematode mitochondria. J Biol Chem.

[B56] Arita M, Suematsu T, Osanai A, Inaba T, Kamiya H, Kita K, Sisido M, Watanabe Y, Ohtsuki T (2006). An evolutionary 'intermediate state' of mitochondrial translation systems found in *Trichinella *species of parasitic nematodes: co-evolution of tRNA and EF-Tu. Nucleic Acids Res.

[B57] Ohtsuki T, Watanabe Y (2007). T-armless tRNAs and elongated elongation factor Tu. IUBMB Life.

[B58] Cui P, Ji R, Ding F, Qi D, Gao HW, Meng H, Yu J, Hu SN, Zhang HP (2007). A complete mitochondrial genome sequence of the wild two-humped camel (*Camelus bactrianus ferus*): an evolutionary history of camelidae. BMC Genomics.

[B59] Davila S, Pinero D, Bustos P, Cevallos MA, Davila G (2005). The mitochondrial genome sequence of the scorpion *Centruroides limpidus *(Karsch 1879) (Chelicerata; Arachnida). Gene.

[B60] Masta SE, Boore JL (2004). The complete mitochondrial genome sequence of the spider *Habronattus oregonensis *reveals rearranged and extremely truncated tRNAs. Mol Biol Evol.

[B61] Lavrov DV, Brown WM, Boore JL (2000). A novel type of RNA editing occurs in the mitochondrial tRNAs of the centipede *Lithobius forficatus*. Proc Natl Acad Sci USA.

[B62] Zhang DX, Hewitt GM (1997). Insect mitochondrial control region: A review of its structure, evolution and usefulness in evolutionary studies. Biochem Syst Ecol.

[B63] Goldstein DB, Schlotterer C (1999). Microsatellites: Evolution and Applications.

[B64] Cameron SL, Whiting MF (2008). The complete mitochondrial genome of the tobacco hornworm, *Manduca sexta*, (Insecta: Lepidoptera: Sphingidae), and an examination of mitochondrial gene variability within butterflies and moths. Gene.

[B65] Yue GH, Liew WC, Orban L (2006). The complete mitochondrial genome of a basal teleost, the Asian arowana (*Scleropages formosus*, Osteoglossidae). BMC Genomics.

[B66] Mayer F, Kerth G (2005). Microsatellite evolution in the mitochondrial genome of Bechstein's bat (*Myotis bechsteinii*). J Mol Evol.

[B67] Zardoya R, Meyer A (1998). Cloning and characterization of a microsatellite in the mitochondrial control region of the African side-necked turtle, *Pelomedusa subrufa*. Gene.

[B68] Arnason U, Johnsson E (1992). The complete mitochondrial DNA sequence of the harbor seal, *Phoca vitulina*. J Mol Evol.

[B69] Arnason U, Gullberg A, Johnsson E, Ledje C (1993). The nucleotide sequence of the mitochondrial DNA molecule of the gray seal *Halichoerus grypus*, and a comparison with mitochondrial sequences of other true seals. J Mol Evol.

[B70] Hoelzel AR, Hancock JM, Dover GA (1993). Generations of VNTRS and heteroplasmy by sequence turnover in the mitochondrial control region of 2 elephant seal species. J Mol Evol.

[B71] Saccone C, Pesole G, Sbisa E (1991). The main regulatory region of mammalian mitochondrial DNA – Structure function model and evolutionary pattern. J Mol Evol.

[B72] Zardoya R, Meyer A (1996). The complete nucleotide sequence of the mitochondrial genome of the lungfish (*Protopterus dolloi*) supports its phylogenetic position as a close relative of land vertebrates. Genetics.

[B73] He Y, Jones J, Armstrong M, Lamberti F, Moens M (2005). The mitochondrial genome of *Xiphinema americanum *sensu stricto (Nematoda: Enoplea): Considerable economization in the length and structural features of encoded genes. J Mol Evol.

[B74] Shao RF, Barker SC, Mitani H, Takahashi M, Fukunaga M (2006). Molecular mechanisms for the variation of mitochondrial gene content and gene arrangement among chigger mites of the genus *Leptotrombidium *(Acari: Acariformes). J Mol Evol.

[B75] De Rijk P, Robbrecht E, De Hoog S, Caers A, Peer Y Van de, De Wachter R (1999). Database on the structure of large subunit RNA. Nucleic Acids Res.

[B76] Peer Y Van de, Caers A, De Rijk P, De Wachter R (1998). Database on the structure of small ribosomal subunit RNA. Nucleic Acids Res.

[B77] Yamazaki N, Ueshima R, Terrett JA, Yokobori S, Kaifu M, Segawa R, Kobayashi T, Numachi K, Ueda T, Nishikawa K, Watanabe K, Thomas RH (1997). Evolution of pulmonate gastropod mitochondrial genomes: Comparisons of gene organizations of *Euhadra*, *Cepaea *and *Albinaria *and implications of unusual tRNA secondary structures. Genetics.

[B78] Farris JS, Kallersjo M, Kluge AG, Bult C (1995). Constructing a significance test for incongruence. Syst Biol.

[B79] Cunningham CW (1997). Can three incongruence tests predict when data should be combined?. Mol Biol Evol.

[B80] Dolphin K, Belshaw R, Orme CDL, Quicke DLJ (2000). Noise and incongruence: Interpreting results of the incongruence length difference test. Mol Phylogenet Evol.

[B81] Yoder AD, Irwin JA, Payseur BA (2001). Failure of the ILD to determine data combinability for slow loris phylogeny. Syst Biol.

[B82] Barker FK, Lutzoni FM (2002). The utility of the incongruence length difference test. Syst Biol.

[B83] Dowton M, Austin AD (2002). Increased congruence does not necessarily indicate increased phylogenetic accuracy – The behavior of the incongruence length difference test in mixed-model analyses. Syst Biol.

[B84] Hipp AL, Hall JC, Sytsma KJ (2004). Congruence versus phylogenetic accuracy: Revisiting the incongruence length difference test. Syst Biol.

[B85] Kluge AG (1989). A concern for evidence and a phylogenetic hypothesis of relationships among Epicrates (Boidae, Serpentes). Syst Zool.

[B86] Felsenstein J (1978). Cases in which parsimony or compatibility methods will be positvely misleading. Syst Zool.

[B87] Cunningham CW, Zhu H, Hillis DM (1998). Best-fit maximum-likelihood models for phylogenetic inference: Empirical tests with known phylogenies. Evolution.

[B88] Barker SC, Murrell A (2002). Phylogeny, evolution and historical zoogeography of ticks: a review of recent progress. Exp Appl Acarol.

[B89] Klompen H, Lekvelshvili M, Black WC (2007). Phylogeny of parasitiform mites (Acari) based on rRNA. Mol Phylogenet Evol.

[B90] Murrell A, Dobson SJ, Walter DE, Campbell NJH, Shao RF, Barker SC (2005). Relationships among the three major lineages of the Acari (Arthropoda: Arachnida) inferred from small subunit rRNA: paraphyly of the parasitiformes with respect to the opilioacariformes and relative rates of nucleotide substitution. Invertebr Syst.

[B91] Miyamoto J, Ishii A, Sasa M (1975). Successful method for mass-culture of house dust mite, *Dermatophagoides pteronyssinus *(Trouessart, 1897). Jpn J Exp Med.

[B92] Kalpaklioglu AF, Ferizli AG, Misirligil Z, Demirel YS, Gurbuz L (1996). The effectiveness of benzyl benzoate and different chemicals as acaricides. Allergy.

[B93] Bischoff E (1988). Méthodes actuelles de quantification des acariens dans l'habitat. Rev Fr Allergol.

[B94] Hart B, Guerin (1990). Ecology and biology of allergenic mites. Mites and Allergic disease.

[B95] Sambrook J, Russel D (1987). Molecular Cloning: a Laboratory Manual.

[B96] Rice P, Longden I, Bleasby A (2000). EMBOSS: The European Molecular Biology Open Software Suite. Trends Genet.

[B97] Altschul SF, Madden TL, Schaffer AA, Zhang JH, Zhang Z, Miller W, Lipman DJ (1997). Gapped BLAST and PSI-BLAST: a new generation of protein database search programs. Nucleic Acids Res.

[B98] Lowe TM, Eddy SR (1997). tRNAscan-SE: A program for improved detection of transfer RNA genes in genomic sequence. Nucleic Acids Res.

[B99] Thompson JD, Higgins DG, Gibson TJ (1994). CLUSTAL-W – Improving the sensitivity of progressive multiple sequence alignment through sequence weighting, position-specific gap penalties and weight matrix choice. Nucleic Acids Res.

[B100] Hall T (1999). BioEdit: a user-friendly biological sequence alignment-editor and analysis program for Windows 95/98/NT. Nucleic Acids Symp Ser.

[B101] Campanella JJ, Bitincka L, Smalley J (2003). MatGAT: An application that generates similarity/identity matrices using protein or DNA sequences. BMC Bioinformatics.

[B102] Bernt M, Merkle D, Ramsch K, Fritzsch G, Perseke M, Bernhard D, Schlegel M, Stadler PF, Middendorf M (2007). CREx: inferring genomic rearrangements based on common intervals. Bioinformatics.

[B103] Zuker M (2003). Mfold web server for nucleic acid folding and hybridization prediction. Nucleic Acids Res.

[B104] De Rijk P, Wuyts J, De Wachter R (2003). RnaViz 2: an improved representation of RNA secondary structure. Bioinformatics.

[B105] Castresana J (2000). Selection of conserved blocks from multiple alignments for their use in phylogenetic analysis. Mol Biol Evol.

[B106] Abascal F, Posada D, Knight RD, Zardoya R (2006). Parallel evolution of the genetic code in arthropod mitochondrial genomes. PLoS Biol.

[B107] Xia XH, Xie Z, Salemi M, Chen L, Wang Y (2003). An index of substitution saturation and its application. Mol Phylogenet Evol.

[B108] Xia X, Xie Z (2001). DAMBE: Software package for data analysis in molecular biology and evolution. J Hered.

[B109] Swofford DL (2003). PAUP*. Phylogenetic Analysis Using Parsimony (*and Other Methods), version 4.0b10.

[B110] Abascal F, Zardoya R, Posada D (2005). ProtTest: selection of best-fit models of protein evolution. Bioinformatics.

[B111] Posada D, Crandall KA (1998). MODELTEST: testing the model of DNA substitution. Bioinformatics.

[B112] Jobb G, von Haeseler A, Strimmer K (2004). TREEFINDER: a powerful graphical analysis environment for molecular phylogenetics. BMC Evol Biol.

[B113] Huelsenbeck JP, Ronquist F (2001). MRBAYES: Bayesian inference of phylogenetic trees. Bioinformatics.

