# Supplementary figures and images for: The complete mitochondrial genome of the house dust mite Dermatophagoides pteronyssinus (Trouessart): a novel gene arrangement among arthropods
Source: BMC Genomics. 2009 Mar 13;10:107. doi: 10.1186/1471-2164-10-107 (PMC2680895; doi:10.1186/1471-2164-10-107)

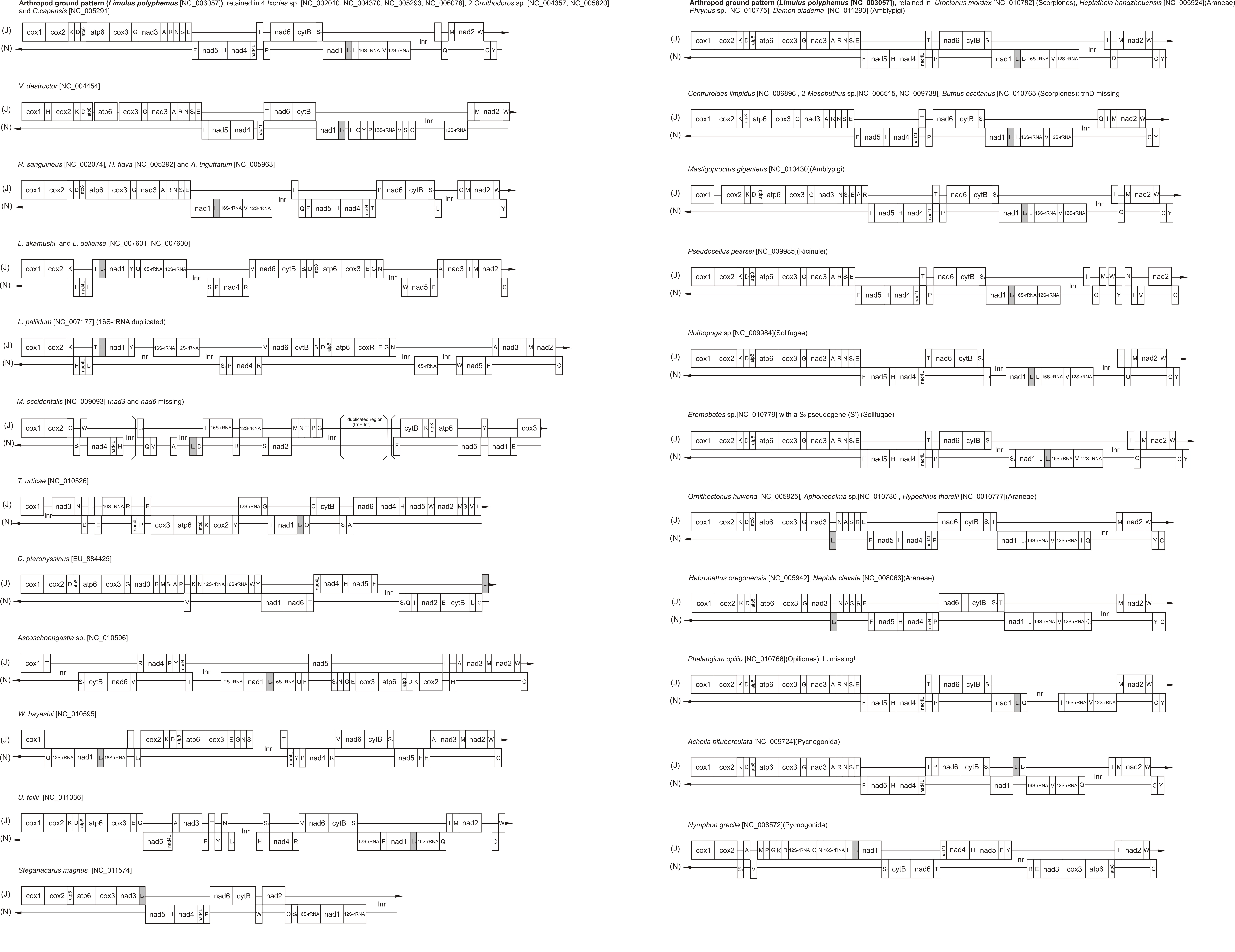

Supplement: Additional file 1 — Mitochondrial genome arrangements of 42 Chelicerata. Graphical linearisation of the mt genomes was done according to [32] (see Fig. 3). Corresponding GenBank accession numbers are between brackets. The position of trnL2 is grey shaded. Protein coding and rRNA genes are abbreviated as in the Abbreviations section; tRNA genes are abbreviated using the one-letter amino acid code. Small non-coding regions (> 50bp) are indicated as gaps between genes. Braces accentuate the duplicated region in the mt genome of M. occidentalis. [file 1471-2164-10-107-S1.jpeg]

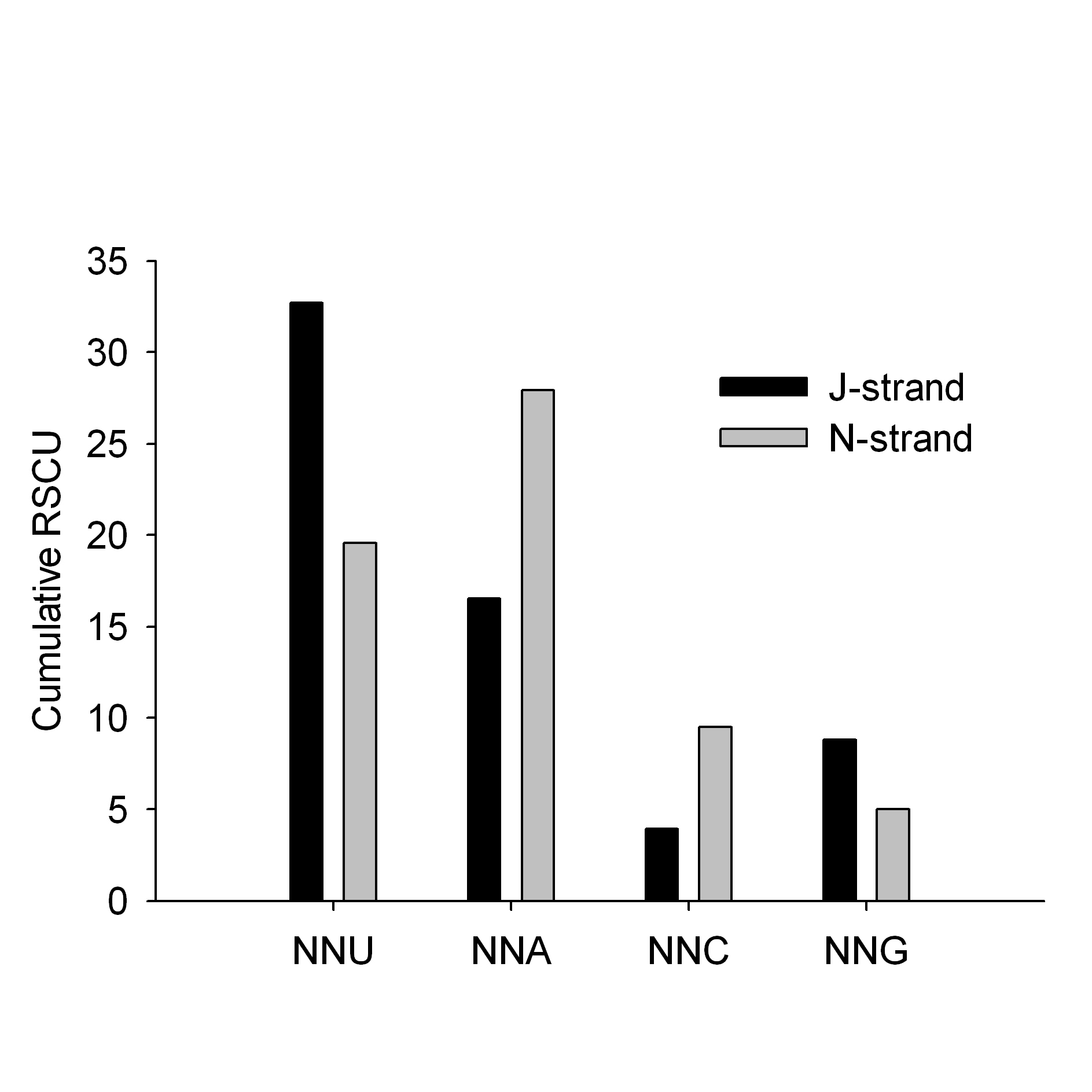

Supplement: Additional file 2 — Across-strand (N and J) comparison of frequencies of codons ending with the same nucleotide. Values on the y-axis represent the sum of Relative Synonymous Codon Usage (RSCU) values (Table 3) of codons ending with the same nucleotide across all codon families (x-axis). [file 1471-2164-10-107-S2.jpeg]

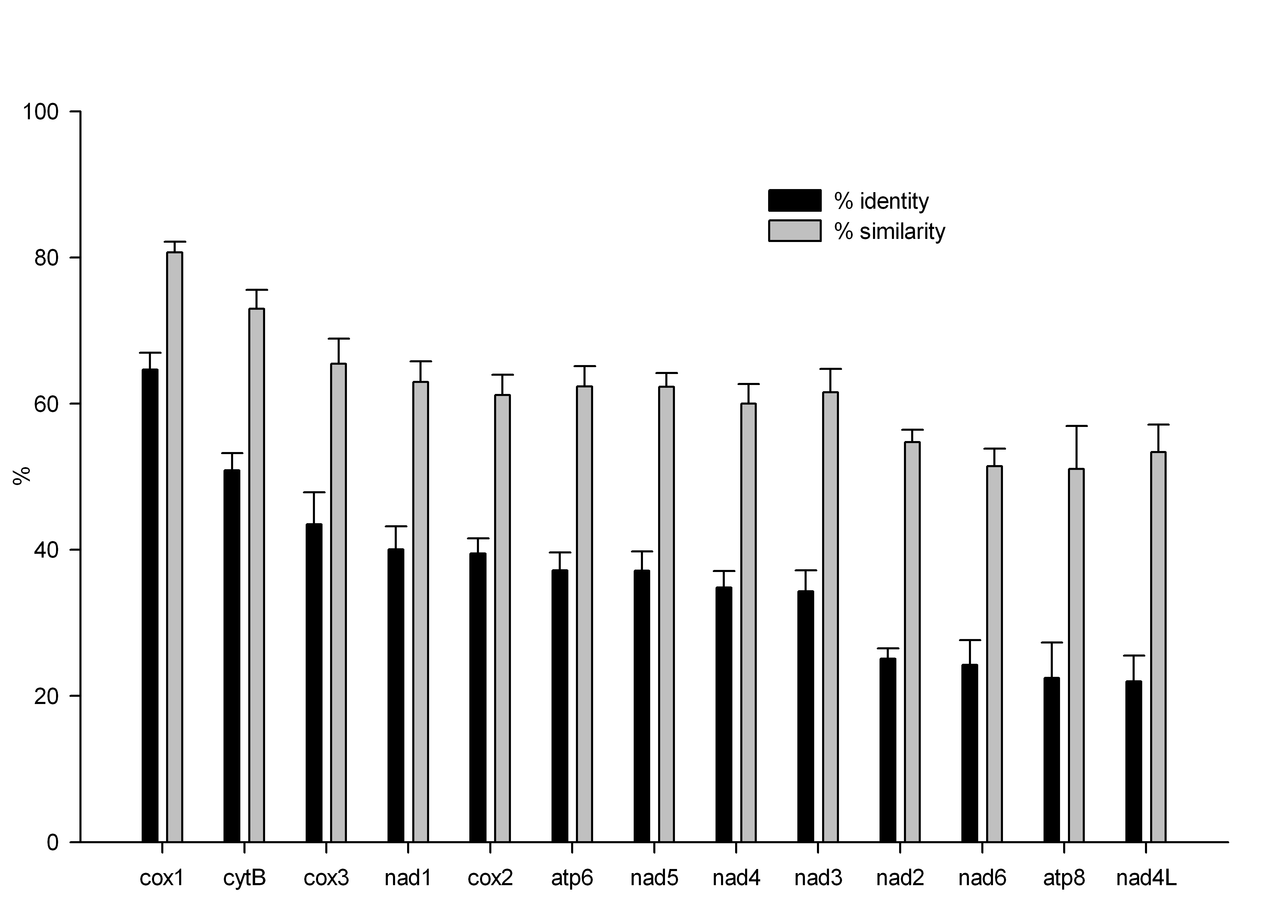

Supplement: Additional file 3 — Average identity and similarity % of mt proteins of D. pteronyssinus. For each protein of D. pteronyssinus, a similarity and identity value was calculated with the corresponding protein of other Acari species (as listed in Table 1), using pairwise global alignment. The obtained values were used to calculate an average identity and similarity % for each protein. [file 1471-2164-10-107-S3.jpeg]

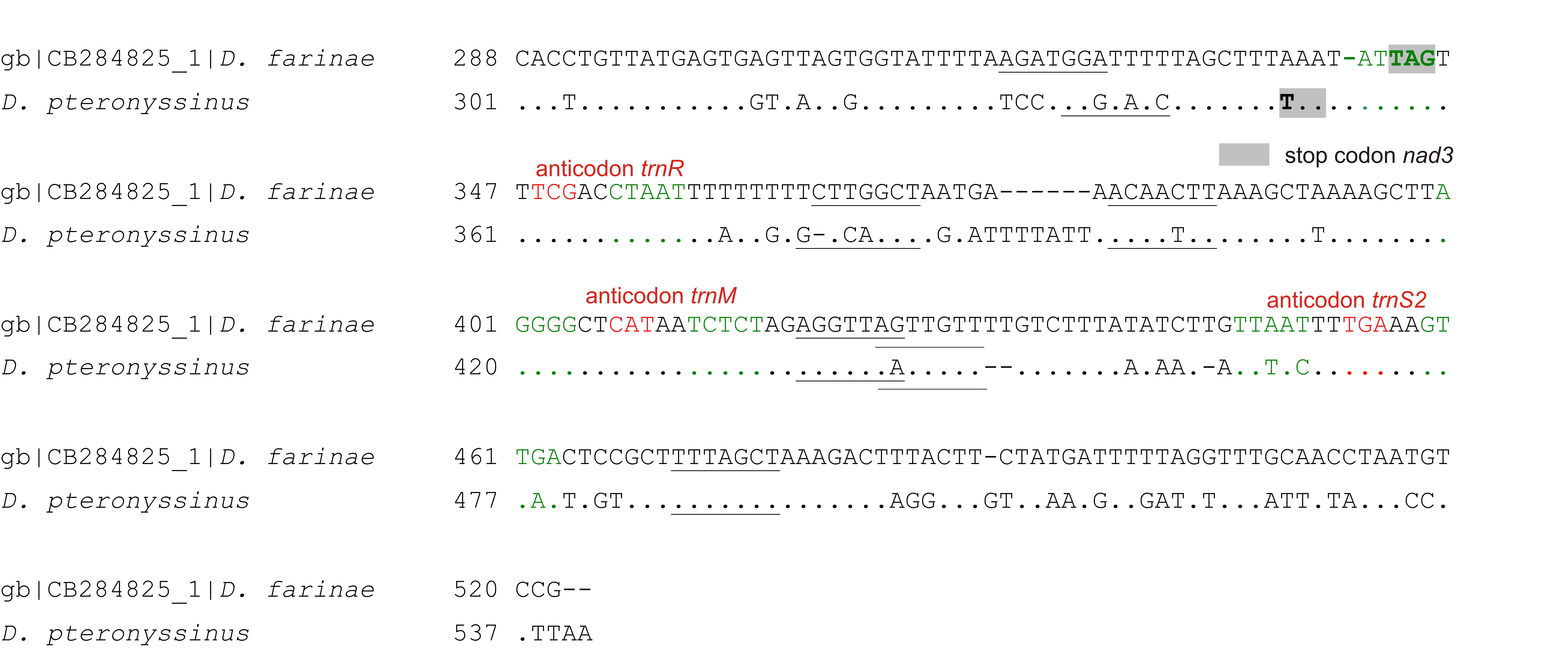

Supplement: Additional file 6 — Alignment of a mt genome fragment containing trnaM, trnaR and trnaS2 of D. pteronyssinus and an EST [GenBank: CB284825] of D. farinae. Anticodons and anticodon stems are red and green respectively. Acceptor-stems of trnR, trnM and trnS2 are underlined. The stop codon of nad3 is grey shaded. [file 1471-2164-10-107-S6.jpeg]
