# Supplementary material for: The complete mitochondrial genome of the house dust mite Dermatophagoides pteronyssinus (Trouessart): a novel gene arrangement among arthropods
Source: BMC Genomics. 2009 Mar 13;10:107. doi: 10.1186/1471-2164-10-107 (PMC2680895; doi:10.1186/1471-2164-10-107)
Supplement: Additional file 7 — Primers and their sequences used to characterise the D. pteronyssinus mt genome. [file 1471-2164-10-107-S7.doc]

| **Primer** | **Sequence (5’-3’)** | **Tm (°C)** |
| --- | --- | --- |
| 12SID-F | TTTTTCTGGCGGTTTATTACC | 58 |
| 12SID-R | CGACTTATCTATCAAAAGAGTGACC | 59 |
| COXI-F | GCTGTTCCTACGGGAGTTAAGG | 61 |
| COXI-R | ACACGCCCTCTTCTCATACC | 59 |
| 12S-F | GCGGTTTATTACCCATTCACAGG | 61 |
| 12S-R | ACGGGCGATATGTACTTTTATTAGG | 63 |
| CYTB-F-Deg | TAWRAARTATCAYTCDGGTTKRATATG |  |
| CYTB-R-Deg | CCWTGAGGACAAATAWSWTTYTGAGG |  |
| CYTB-F | CGATTCAAGGTGGGTTGG | 60 |
| CYTB-R | GATATTGACCACGGTTGAATTATGC | 62 |
| DP-Ms-F | TGCTAAAGCAAGAGAGAACTTTT | 58 |
| Dp-Ms-R | CCCCTTCAAACCACAACACT | 60 |
